# Repeating Nontectonic Seasonal Stress Changes and a Possible Triggering Mechanism of the 2019 Ridgecrest Earthquake Sequence in California

**DOI:** 10.1029/2021JB022188

**Published:** 2021-09-29

**Authors:** Jeonghyeop Kim, William E. Holt, Alireza Bahadori, Weisen Shen

**Affiliations:** ^1^ Department of Geosciences Stony Brook University Stony Brook NY USA

## Abstract

Here we characterize the 13‐year history of nontectonic horizontal strain anomalies across the regions surrounding Ridgecrest, CA, using cGPS data from January 2007. This time‐dependent model reveals a seasonality in the nontectonic strain anomalies and the associated Coulomb stress changes of ∼±0.5–2 kPa. In the area surrounding the epicenters of the 2019 Ridgecrest earthquake sequence of July, we find that the seasonal preseismic Coulomb stress changes peaked every early summer (May and June) during the last 13 years including during June 2019, a month prior to the large events. In addition, our statistical tests confirm that more strike‐slip earthquakes (M_w_ ≥ 2) occur during times when seasonal stress changes are increasing on right‐lateral faults in comparison with times when stresses are decreasing. These results suggest that the timing of the 2019 Ridgecrest earthquakes may have been modulated by nontectonic seasonal stress changes. The dynamic source of the seasonal nontectonic strain/stress anomalies, however, remains enigmatic. We discuss a possible combination of driving forces that may be attributable for the seasonal variations in nontectonic strain/stress anomalies, which captured in cGPS measurements.

## Key Points

- Our horizontal cGPS analysis for the last 13 years shows seasonal nontectonic strain anomalies in regions surrounding Ridgecrest, CA
- The associated Coulomb stress changes peak every early summer including June 2019, during the month prior to the 2019 Ridgecrest earthquakes
- More earthquakes have occurred in the Ridgecrest region when the stress changes favor the failure of right‐lateral faults

## 1 Introduction

Dense spatial and temporal coverage of continuous global positioning system (cGPS) measurement enables rigorous investigations of time‐dependent surface strain anomaly patterns in California. Observed by cGPS, the seasonality in the crustal nontectonic strain and the associated stress changes are attributable to the Earth’s elastic response (Farrell, 1972) to time‐varying loading sources on the surface, such as hydrologic loads (e.g., Argus et al., 2014), atmospheric pressure (e.g., Gao et al., 2000), and thermoelastic loads (e.g., Ben‐Zion & Allam, 2013; Prawirodirdjo et al., 2006). Furthermore, studies have reported that cGPS measurements can also capture multiyear variations in crustal strain due to drought and anomalously heavy precipitation in California (e.g., Argus et al., 2017; Borsa et al., 2014; Hammond et al., 2019; Kim et al., 2021; Klein et al., 2019; Silverii et al., 2020).

Using cGPS data of the Plate Boundary Observatory (PBO; Herring et al., 2016; now Network of the America [NOTA]), recent studies show statistical correlations between seasonal stress changes and earthquakes from declustered catalogs, which suggest such stress changes may modulate seismicity rates within the plate boundary zone in California (e.g., Johnson et al., 2017a, 2017b; Kreemer & Zaliapin, 2018). Kraner et al. (2018) showed that the seasonal Coulomb stress changes peaked in the region surrounding the 2014 M_w_ 6.0 South Napa earthquake of August 24 in mid‐late summer, including just a month prior to the event. Their analysis was consistent with the hypothesis that this large event was triggered by nontectonic seasonal stress changes. Johnson et al. (2017a) also showed a possible seasonality in 137 large historic earthquakes (≥M 5.5 between 1781 and 2012).

Up to 2019, the 2014 South Napa earthquake was the only large inland event (M_w_ ≥ 6) in California covered by the rigorous measurements of PBO network after completion of its installation in 2008. The 2010 M_w_ 7.2 El Mayor‐Cucapah earthquake, Mexico, occurred at the periphery of PBO coverage. It is thus challenging to quantify a rigorous preseismic strain pattern there, owing to the asymmetric distribution of cGPS stations.

In July 2019, two large earthquakes (M_w_ ≥ 6) occurred in Ridgecrest, CA, within ∼35 hr of each other (e.g., Hough et al., 2020). The occurrence of the 2019 Ridgecrest earthquake sequence opens an additional opportunity to quantify the state of preseismic strain prior to these large events and to test Kraner et al.’s hypothesis that a large earthquake can be triggered by seasonal stress changes. Here, based on cGPS measurements of NOTA, we characterize the 13‐year history of the preseismic, nontectonic strain anomaly patterns and the associated stress changes, leading up to the 2019 Ridgecrest earthquake sequence in regions surrounding Ridgecrest, CA. We also perform statistical tests to investigate a possible correlation between small earthquakes from a declustered catalog (M_w_ ≥ 2) and our nontectonic stress anomaly estimates.

## 2 Data and Methods

### 2.1 cGPS Data Sets and the Total Stress Estimates That Consist of Time‐Dependent Nontectonic Stress Changes and Steady‐State Tectonic Stress Loading Rates

**Figure 1:**
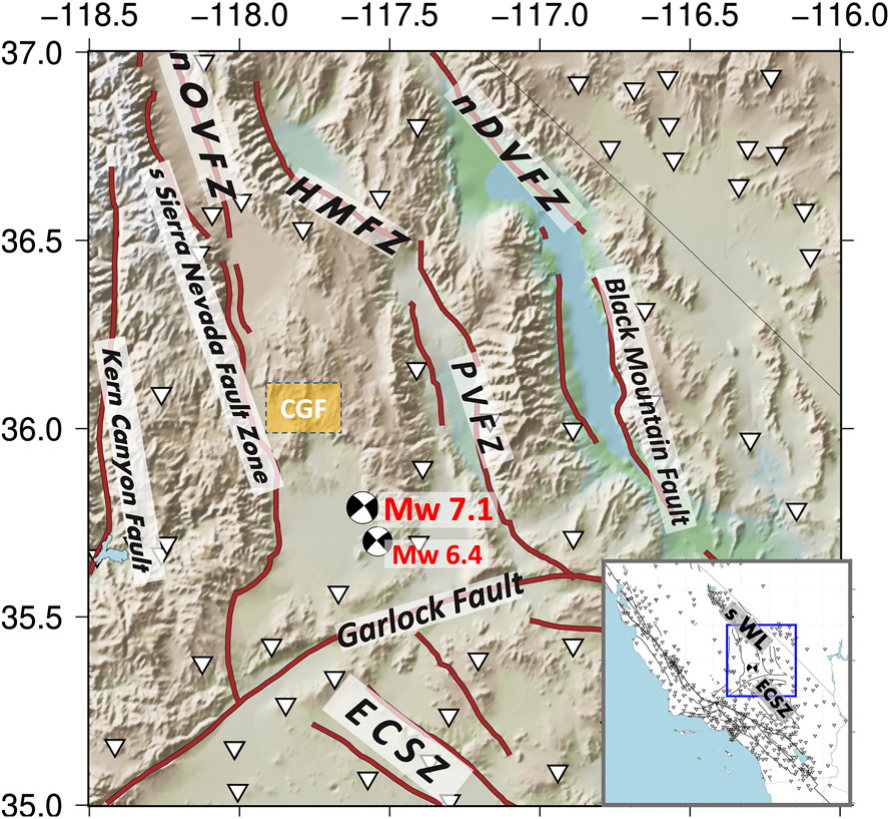
A map of the ECSZ‐WL transition zone in California south of latitude 37.0°N. The background is topography (ETOPO 1; Amante & Eakins, 2009). cGPS stations we use for the analysis are indicated as the inverted white triangles. The focal mechanisms of the 2019 Ridgecrest earthquake sequence are shown, where compressional quadrants are shaded in black. The blue box in the inset map shows the ECSZ‐WL transition zone in a large‐scale view. Fault zones and provinces are labeled, and the dark reddish‐brown lines are the major faults in the transition zone provided by Jennings (1994). nOVFZ, northern Owens Valley Fault Zones; HMFZ, Hunter Mountain Fault Zones; PVFZ, Panamint Valley Fault Zones; nDVFZ, northern Death Valley Fault Zones; sWL, southern Walker Lane belt; ECSZ, Eastern California Shear Zone.

We use the cGPS level‐2 position time series data sets processed by the Nevada Geodetic Laboratory (NGL [http://geodesy.unr.edu]; Blewitt et al., 2018). These data sets are defined with respect to the IGS14 frame of reference (Altamimi et al., 2016). We first analyze 908 vertical position time series in California and western Nevada, and we eliminate 230 stations that may be affected by poroelastic processes. To distinguish such stations, we follow the methodology of Argus et al. (2014). If a station is in minimum vertical position during the wet seasons (e.g., winter in California), we infer that the station shows the elastic response to the maximum surface hydrologic loads. If a poroelastic effect is dominant for a station, the pattern is opposite: excessive water from the precipitation fills up pore spaces in sediments, causing the maximum uplift during the wet seasons. Thus, we eliminate stations that show maximum vertical positions during the wet season of November–April (Kim et al., 2021). We also omit stations that show subsidence rates larger than 1 mm/yr during the severe drought (2015–2021) in California, assuming that the high subsidence rates represent anthropogenic signatures captured by cGPS (e.g., groundwater extraction; Argus et al., 2014). If no measurement is available during the severe drought for a station, we take the conservative approach of eliminating the station. We present the retained 678 cGPS data in the inset map in Figure 1.

Using metadata provided by the NGL (http://geodesy.unr.edu/NGLStationPages/steps.txt), we also find discontinuous steps pertaining to equipment changes in cGPS horizontal position time series data. To correct these offsets, we follow the methodology of Johnson et al. (2021). For instance, we first model a discontinuous step with the difference between the median positions of the 14 days before and after the step, and then subtract the modeled step from the position time series. We carefully investigated the offset‐corrected position time series of cGPS measurements within the Ridgecrest region (Figure 1) and we found that the algorithm properly eliminates equipment‐related steps (Figure S1). In addition, we identified and omitted a large unlisted step (Figure S1c) and outliers that may introduce artifacts in our strain estimates.

Following Kim et al. (2021), we quantify the 13‐year (January 2007–June 2019) history of horizontal strain patterns and the associated stress changes for California and western Nevada based on the corrected 678 cGPS data sets. For each month of the 13 years, we invert horizontal displacement measurements (cGPS) for continuous displacement and strain field estimates. We convert the horizontal strain field estimates into a horizontal stress field, assuming that the shallow portion of the Earth’s crust is elastic and isotropic. After this we quantify Coulomb stress changes on expected vertical strike‐slip orientations (see Section 1 in Text S1 for details). This time‐dependent horizontal strain/stress model captures displacements up to the end of June, just prior to the July 4 and 5, 2019 Ridgecrest foreshock and mainshock. Our interest is the preseismic evolution of the horizontal strain and stress patterns in the Ridgecrest region. One advantage of our methodology is that we can “decompose” the total horizontal strain into time‐dependent nontectonic strain anomalies (inferred from cGPS) and a steady‐state “tectonic” strain (inferred from the UCERF3 GPS velocity model; Field et al., 2014; Parsons et al., 2013; see Section 1 in Text S1). Thus, we investigate a possible influence of the preseismic evolution of the stress changes on seismicity rates, while taking into account both the steady‐state tectonic stress loading rates (e.g., Del Pardo et al., 2012; Smith‐Konter et al., 2020) and the nontectonic stress variations (e.g., Kraner et al., 2018). Note that we call the sum of these two types of stress estimates the total stress changes. These total stress changes vary spatially (within 0.1° × 0.1°–0.3° × 0.3° grid cells) and temporally (defined monthly).

### 2.2 Definitions of the Expected Number of Earthquakes and the Excessive Number of Earthquakes From a Declustered Earthquake Catalog

Based on the total stress change estimates (e.g., King et al., 1994), we define a “stressing period,” within a particular grid cell, as a time interval in which each successive time step (a month) reaches a new Coulomb stress high in comparison to all previous time steps. Once a Coulomb stress value drops below the previous peak, we define the total stress time series as having entered a “relaxing period” within that grid cell (Figure S2).

One of our null hypotheses is that earthquakes occur regardless of seasonal stress changes in regions surrounding Ridgecrest, CA. That is, the rate of seismicity is expected to be independent of seasonal stress changes and it will thus be a constant rate within any given area. For instance, within a chosen grid cell, if 40% of the total time is occupied by “stressing periods,” we would expect 40% of all the earthquakes to occur during those periods. Likewise, 60% of all the earthquakes would occur during time intervals occupied by “relaxing periods.” Therefore, if there were 100 earthquakes within the chosen grid cell, our expected number of earthquakes during the “stressing period” is 40. Note that this expected number of earthquakes varies spatially depending on two factors: (a) the slope of the steady‐state tectonic loading rates and (b) the amplitude of the nontectonic stress changes (Figure S2).

After determining the expected number of earthquakes within each of the grid cells in a region of interest, we quantify the excessive number of earthquakes during the “stressing period” for the region by computing

$$N_{ex}=\;\frac{\left(\sum_{i}N_{\text{obs}}-\sum_{i}N_{\text{expected}}\right)}{\sum_{i}N_{\text{expected}}}$$

where *N*_*ex*_ is the excessive number of earthquakes during the “stressing period” for the region of interest; *i* is the total number of grid cells within the region; *N*_obs_ is the actual number of earthquakes during the stressing period for each grid cell; *N*_expected_ is the expected number of earthquakes for each grid cell (Cochran et al., 2004; Johnson et al., 2017a).

**Figure 2:**
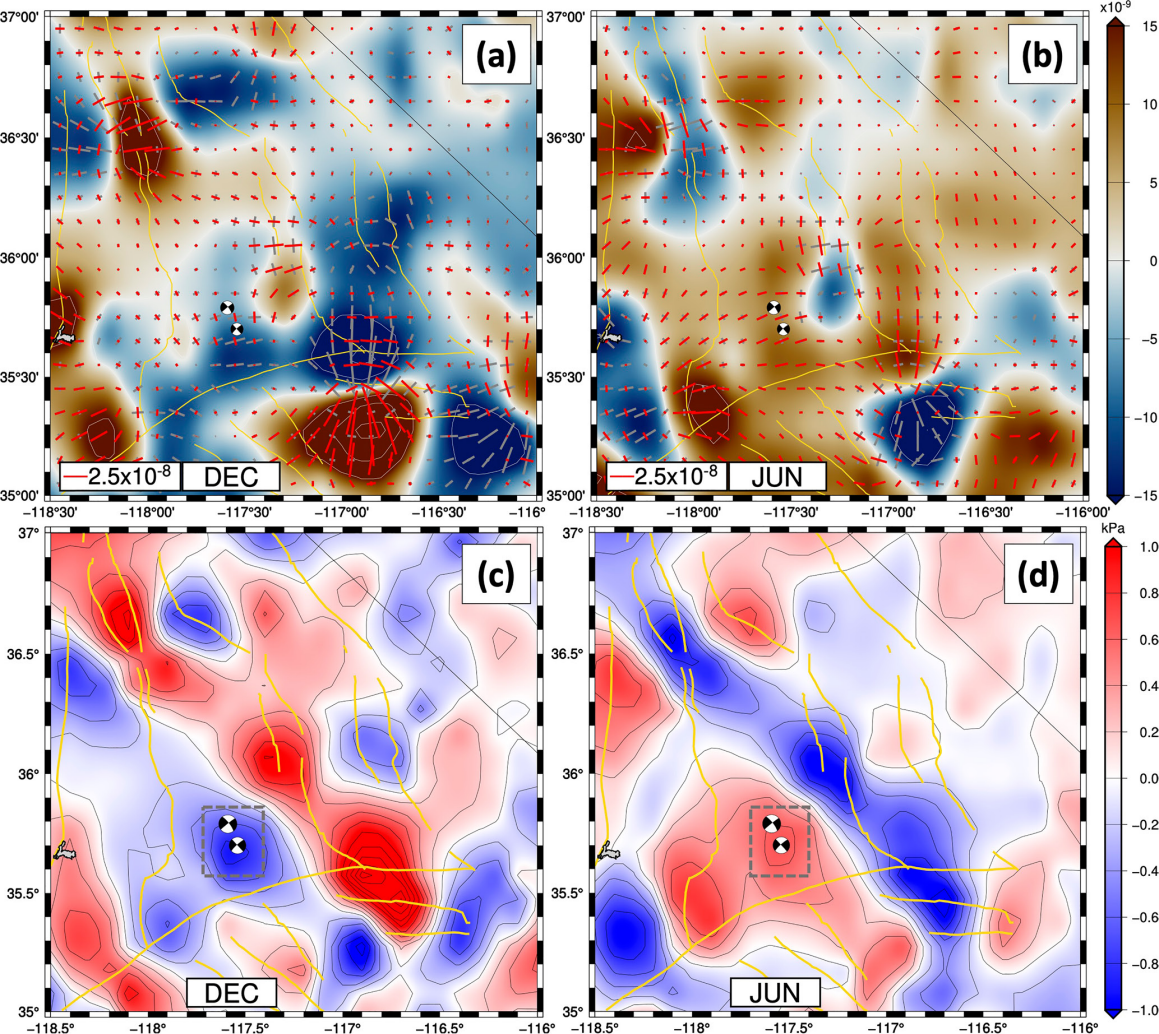
Short‐wavelength, high‐amplitude stacked nontectonic strain solutions over years between 2007 and 2019 (except 2010) (a) for December and (b) for June. Principal axes of the nontectonic strain are shown: Gray bars are contractional and red bars are extensional. The background is dilatational strain (red is extensional). Major strike‐slip fault systems presented in Figure 1 are presented here as yellow solid lines. Stacked Coulomb stress changes associated with the nontectonic strain anomalies are shown for (c) December and (d) June. The warmer color is positive Coulomb stress changes on right‐lateral vertical fault geometries with strike patterns that follow the trends of right‐lateral faults. The dashed boxes in (c) and (d) indicate the Ridgecrest area (892 km^2^) surrounding the epicenters of 2019 Ridgecrest earthquakes where we show the 13‐year time series of seasonal Coulomb stress changes in Figure 3.

We carry out our statistical tests for two target areas. The first area is an 892 km^2^ area surrounding the epicenters of the 2019 Ridgecrest earthquakes (the boxed area in Figures 2c and 2d). The second target area is the entire region shown in Figure 1 (The transition zone between the Eastern California Shear Zone and southern Walker Lane belt, e.g., DuRoss et al., 2020). Hereafter we will refer to these two target areas as the Ridgecrest area and the ECSZ‐WL transition zone, respectively.

For the Ridgecrest area (the first target area), we use one grid cell (*i* = 1) of 0.3° × 0.3° dimension in latitude and longitude, and its spatially averaged (but varying over time) stress value over the cell for our statistical tests (see Section 2.3 below). For the Ridgecrest area, we use all 33 earthquakes (M_w_ ≥ 2; between 2008 and 2018) from the declustered SCSN catalog (the Southern California Seismic Network catalog; Hutton et al., 2010) provided by Zaliapin and Ben‐Zion (2020). Most of these events are strike‐slip‐faulting earthquakes within the Ridgecrest area. For this area we are able to extend our analysis for a longer time span using 141 SCSN declustered earthquakes (M_w_ ≥ 2 between 1981 and 2018) and by using a stacked (averaged) solution. To obtain the stacked solution, we overlapped the solutions for years between 2007 and 2019 (except 2010) and then took the average for each month. For this extended analysis for the longer time span, we assume that the monthly values within the stacked solution are representative of the expected seasonal strain changes over the last 38 years (see Section 3.1).

For the ECSZ‐WL transition zone (the second target area), we use 120 grid cells (*i* = 1,2, … ,120) of 0.2° × 0.2° dimension of latitude and longitude. For the ECSZ‐WL area, grid cells of 0.3° × 0.3° dimension are too large to accurately capture spatial variations in strain. That is, many of these larger grid cells contain the boundary between positive and negative Coulomb stress regimes. On the other hand, the size of 0.1° × 0.1° cells is too small compared to the spatial distribution of NOTA. However, for our stacked solution over a decade (see Section 3.1), we have a higher confidence in the monthly averaged stress estimates. Therefore, for the stacked estimates we use 480 grid cells (*i* = 1,2, … ,480) of 0.1° × 0.1° dimension of latitude and longitude for our statistical tests. We tested 0.2° × 0.2° grid cells for the stacked solution and found the results unchanged. Note that if there was no earthquake within a grid cell, our algorithm skips the grid cell in calculating *N*_*ex*_.

For the seismic events used in the ECSZ‐WL transition zone, we take extra steps to select strike‐slip earthquakes because there exist oblique and normal faults in the ECSZ‐WL transition zone (e.g., Kern Canyon Fault; see Figure 1 in Bawden et al. [1999]; eastern Sierra range front normal fault in Owen’s Valley; see Figure 1 in Johnson et al. [2017b]). It is important to note that our Coulomb stress change estimates are resolved on vertical right‐lateral strike‐slip fault orientations using the three horizontal components of nontectonic strain. Therefore, to investigate whether variations in Coulomb stress on vertical strike‐slip faults have an influence on the timing of strike‐slip earthquakes, it is optimal to include only strike‐slip events in the test.

Based on the declustered SCSN catalog (Zaliapin & Ben‐Zion, 2020), we search for the corresponding focal mechanism solutions (Hauksson et al., 2012; Yang et al., 2012; see Southern California Earthquake Center. Caltech. Data set. https://doi.org/10.7909/C3WD3xH1). We only use strike‐slip focal mechanisms that have rakes greater than 165° or less than −165° (*N* = 227; M_w_ ≥ 2; between 2008 and 2018). Furthermore, since our Coulomb stress change estimates are resolved on right‐lateral fault orientations, we exclude events that occurred on and surrounding the left‐lateral Garlock Fault (*N* = 36). Lastly, we eliminate the earthquakes that occurred in the Coso geothermal field (CGF) region, owing to the possibility that those events may be affected by a different triggering mechanism (*N* = 31; e.g., Feng & Lees, 1998; Fialko & Simons, 2000; Hauksson & Unruh, 2007; Trugman et al., 2016). We are also less confident about our steady‐state tectonic stress model in the CGF region.

Even though our optimal test involves a comparison of strike‐slip earthquakes and the Coulomb stress changes resolved on strike‐slip faults, we should expect that there will be cases where the Coulomb stress change for non‐strike‐slip faults (normal and oblique‐normal) will be positively correlated with the Coulomb stress changes for the strike‐slip faults. The positive correlation will occur when the *T*‐axes of the normal and oblique‐normal faults are close to parallel to the *T*‐axes of the right‐lateral strike‐slip faults. This is generally the case in the ECSZ‐WL transition zone region. Thus, we performed the same statistical tests using all of the earthquakes from the original declustered SCSN catalog (*N* = 611; Zaliapin & Ben‐Zion, 2020) without selecting strike‐slip focal mechanisms. That is, we use all types of earthquakes, which are primarily strike‐slip, oblique‐normal, and normal events, within the ECSZ‐WL transition zone for the last 13 years (Cheng & Ben‐Zion, 2020). We present this result in Section 3.2 as well.

For the ECSZ‐WL transition zone, we lastly extend our analysis for the years between 1981 and 2018 using the stacked (averaged) solution. We have 618 declustered strike‐slip focal mechanisms during this time span, but we only use 499 solutions out of the 618 after we eliminate 119 events that occurred either within the CGF region or on and surrounding the Garlock fault.

### 2.3 Statistical Tests of Null Hypotheses

For each of the two target areas (Ridgecrest and ECSZ‐WL), we perform three different hypothesis tests. Our first null hypothesis is that earthquakes occur with a constant rate, regardless of seasonal stress changes. If the null hypothesis is true, the excessive number of earthquakes during the “stressing period” is expected to be 0% (*N*_obs_ = *N*_expected_) for each grid cell. To determine whether we can accept or reject the null hypothesis, we first place declustered earthquakes for each cell in the exact time when they occurred. We then count how many earthquakes actually occurred during the “stressing period.” Finally, we calculate the excessive number of earthquakes, *N*_*ex*_, during the stressing period.

In order to take into account the formal uncertainties in the total Coulomb stress changes, we repeat this process 100,000 times, in which we perturb the total stress change value by adding random errors within each realization (Movie S1). To account for temporal correlations, we apply a low‐pass filter to a white random noise that follows a Gaussian distribution with the formal standard errors in stress changes (see Section 3 in Text S1). Lastly, we compare the probability function of *N*_*ex*_ obtained from the 100,000 bootstrapped realizations with the *N*_*ex*_ = 0%, given the assumption that the first null hypothesis is true.

We next perform the test for the second null hypothesis: earthquakes occur entirely randomly in time, regardless of seasonal stress changes. For this test, we allow declustered earthquakes to occur randomly in time (their locations are never altered) within each grid cell and calculate *N*_*ex*_ during the stressing period. We repeat this calculation 100,000 times, adding a random error to the total stress changes for each calculation as we did for the first hypothesis test (Movie S1). Note that both the errors in stress, added within each bootstrap realization, and the randomness of timing of earthquake occurrences influence the second probability function of *N*_*ex*_. To determine whether we can accept or reject this second null hypothesis, we compare this second probability function *N*_*ex*_ with the observed excessive number of earthquakes obtained assuming our Coulomb stress change estimates are errorless. We will call this second hypothesis test a random test hereafter.

Lastly, we perform the most stringent test by comparing the two probability functions, both of which account for errors in the stress model, to test the third null hypothesis that the observed excessive number of earthquakes during the stressing period can be explained by earthquakes occurring randomly in time. The overlapping areas between the two probability functions represent the probability that a random process can explain the observations, with a comprehensive consideration of the uncertainties in our stress estimates for both probability functions.

## 3 Results

### 3.1 Seasonally Repeating Nontectonic Strain Anomalies and the Associated Coulomb Stress Changes in Regions Surrounding Ridgecrest, CA

The time‐dependent horizontal nontectonic strain model shows that the anomaly patterns seasonally repeat in space and time over the last 13 years in the entire ECSZ‐WL transition zone. In Supporting Information S1, we present additional animations of the entire 13‐year history of nontectonic strain anomalies and the associated Coulomb stress changes (Movies [Link], [Link], [Link]).

**Figure 3:**
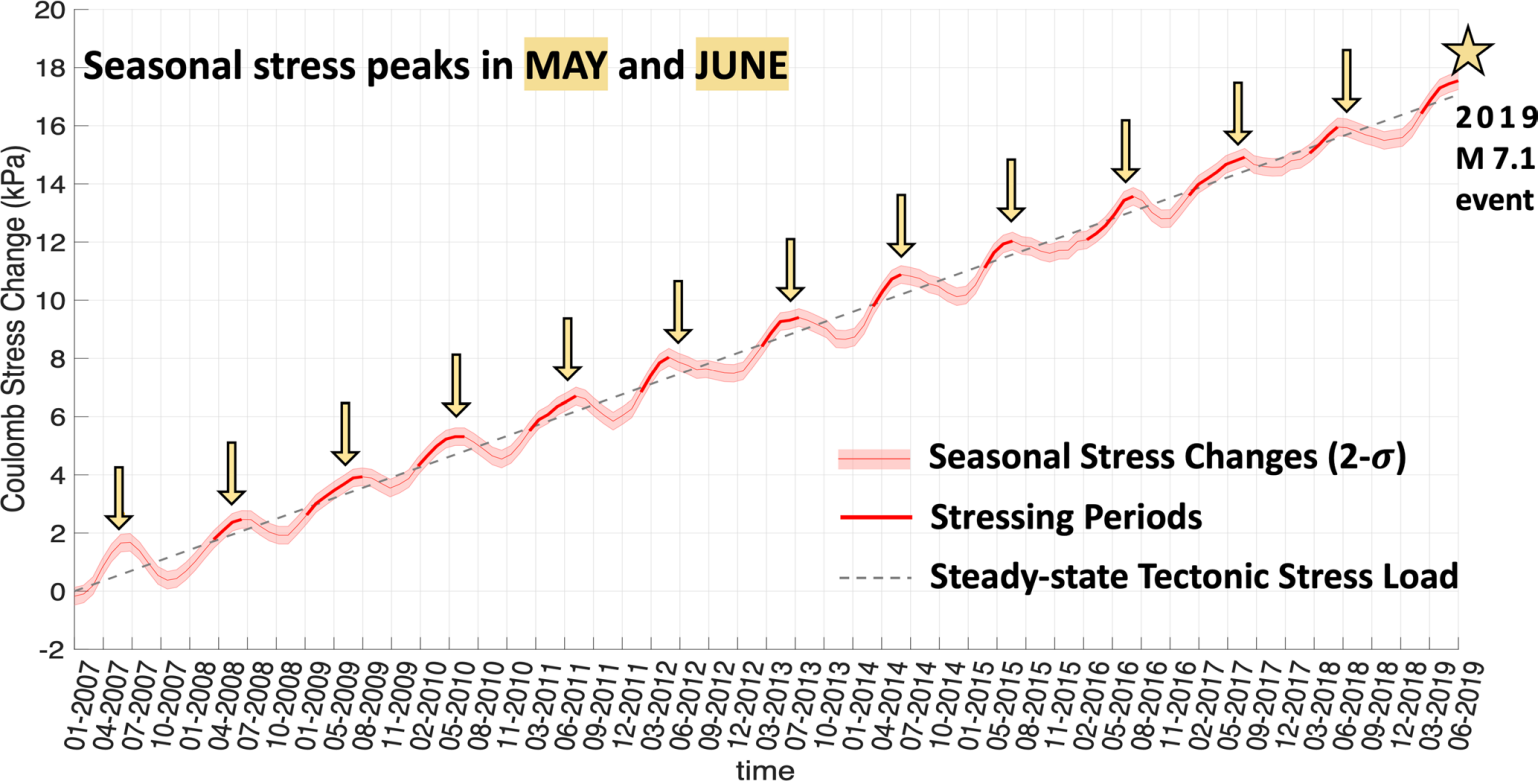
A time series of the nontectonic Coulomb stress changes in the Ridgecrest area surrounding the epicenters of the 2019 Ridgecrest earthquakes (red line with 2 sigma errors). The seasonal Coulomb stress changes are superimposed onto the steady‐state tectonic stress loading rate of 1.4 kPa/yr, which is shown as the gray dashed line. Both the seasonal and tectonic Coulomb stress changes are calculated on a vertical right‐lateral fault geometry striking ∼140° within the Ridgecrest area. The thicker red line indicates “stressing period.” The yellow arrows point to every June over the last 13 years.

We first analyze the seasonally repeating strain anomalies within the 892 km^2^ Ridgecrest area in each year between 2007 and 2019. Contractional principal axes of horizontal strain anomalies within the area tend to align in approximately E‐W directions in each December and January between 2007 and 2019 (Movie S2). On the other hand, within the same area during the May and June time period our model tends to predict extensional strain principal axes aligning along the E‐W directions for the last 13 years. The magnitudes of the seasonally repeating nontectonic strain anomalies are ∼± 2.5 × 10^−8^. These seasonal variations in horizontal strain produce Coulomb stress changes of ∼0.7 kPa (Movie S3) on vertical right‐lateral faults striking ∼140° in the Ridgecrest area. The seasonal Coulomb stress changes peak every May and June (Figure 3), including June 2019, during the month prior to the 2019 Ridgecrest earthquakes.

Across the ECSZ‐WL transition zone, we detect many other places where notable seasonal variations in horizontal strain anomalies reliably repeat over the last 13 years. The southern Sierra Nevada Fault Zone (SNFZ), northern Owen’s Valley Fault Zone (OVFZ), and the Panamint Valley Fault Zones (PVFZ) tend to experience fault‐normal extensional nontectonic strains of 1–4 × 10^−8^ during the winter (December–March). Fault‐normal contractional nontectonic strains of similar magnitude take place during the summer (June–September) on these same fault zones. These seasonally repeating horizontal nontectonic strain anomalies produce Coulomb stress increases of 1–2 kPa on the vertical right‐lateral strike‐slip faults in the winter through the process of unclamping and 1–2 kPa of Coulomb stress decrease in the summer through the process of fault clamping.

The Hunter Mountain Fault and the Kern Canyon Fault north of 36°N show contractional dilatational strain anomalies and associated negative Coulomb stress changes during the winter. These same faults show extensional dilatational strain anomalies and associated positive Coulomb stress changes during the summer. This timing is opposite to that observed on the southern SNFZ, northern OVFZ, and the PVFZ.

The horizontal strain/stress anomalies at any given time have large uncertainties due to errors in cGPS measurements and the inherent spatial aliasing of the NOTA (Movie S4). There are variations in space and amplitude of the nontectonic seasonal strain/stress anomaly estimates over years, depending on the intensity of the precipitation in California (Figure S3; Kim et al., 2021). Nonetheless, we find that the anomaly patterns are reliably similar to one another for all years across the ECSZ‐WL transition zone, including the Ridgecrest area. Therefore, we stack all solutions between 2007 and 2019 (excluding a year period following the 2010 M_w_ 7.2 El Mayor‐Cucapah earthquake, since it has a large tectonic transient (Klein et al., 2019) and our interest is to investigate the nontectonic component in the strain anomalies) to obtain 12 stacked monthly solutions (Figure 2). We also present animations of these stacked seasonal nontectonic strain/stress changes in Movies [Link], [Link], [Link]. We propagate the uncertainties in the strain fields when computing the stacked solution. The errors for the stacked solution are decreased by roughly one over the square root of the number of years (11–12 years; compare Movie S4 with Movie S7).

### 3.2 Statistical Tests of Significance of the Seasonal Stress Changes on Earthquake Occurrences in the Ridgecrest Area and the ECSZ‐WL Transition Zone

In performing the three hypothesis tests, we first use the full 13‐year history of total Coulomb stress change estimates. For the Ridgecrest area, our steady‐state tectonic model predicts a constant tectonic (Coulomb) stress loading rate of 1.4 kPa/yr on the right‐lateral fault geometry (∼140°). We superimposed the seasonally repeating nontectonic Coulomb stress changes (resolved on the same 140° vertical right‐lateral fault geometry) onto the linearly increasing tectonic stress loading estimate. This time series of the total stress changes for the Ridgecrest area reveals that June 2019, a month prior to the 2019 Ridgecrest earthquake sequence, falls into the “stressing period.”

**Figure 4:**
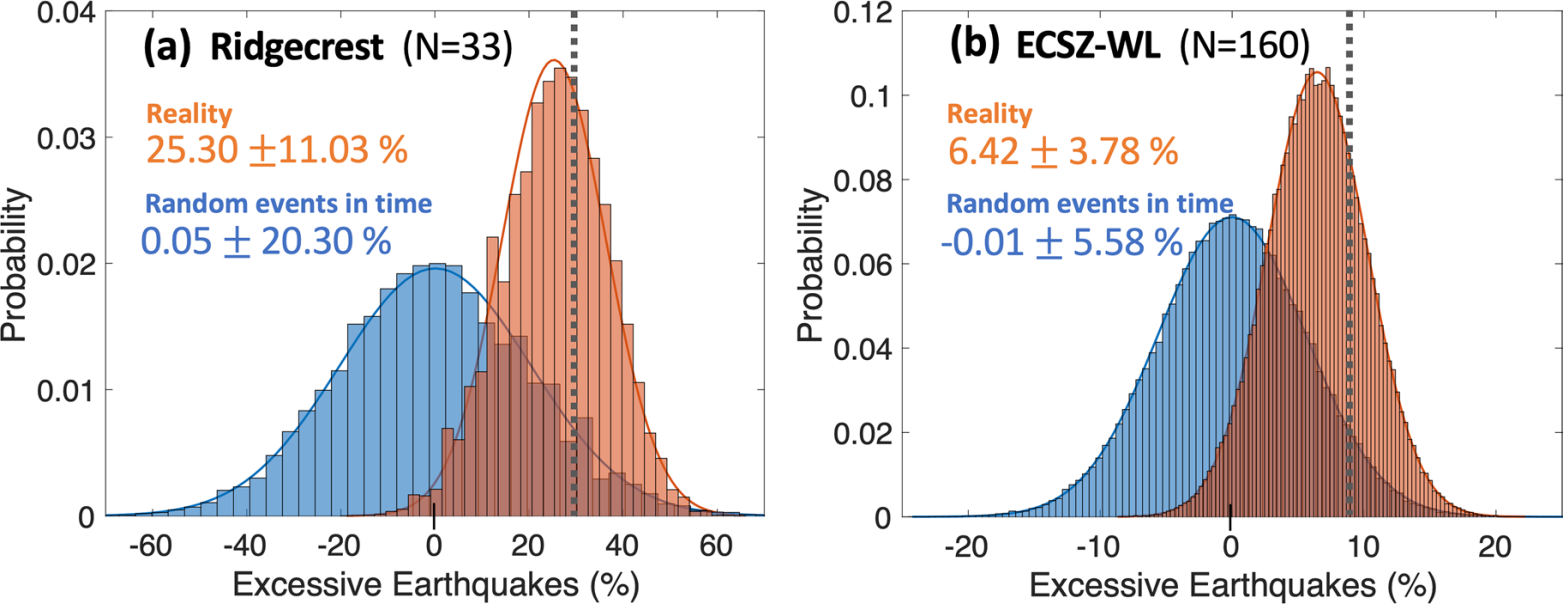
Statistical test results. Probability Density Functions (PDFs) of declustered earthquake occurrences between January 2008 and December 2018 for (a) the Ridgecrest area surrounding the epicenters of the 2019 Ridgecrest earthquake sequence and for (b) the entire ECSZ‐WL transition zone shown in Figure 1. Each of these PDFs is plotted as a function of excessive number of earthquakes (%) during the stressing period. The blue curves are obtained by distributing declustered events randomly in time, while the orange curves are obtained by distributing declustered events in the original time of their occurrences. Both PDFs take into account model errors in stress (see the main text for explanation). Mean values and standard deviations are presented for each test. The dotted gray vertical lines indicate the excessive number of earthquakes (%) obtained assuming our stress model is errorless (28.6% for [a] and 9.1% for [b]).

For the Ridgecrest area, our first hypothesis test reveals that 25.3% ± 11.0% more earthquakes (declustered; M_w_ ≥ 2) occurred during the “stressing period” compared to the “relaxing period” (orange curve in Figure 4a) while we expect 0% more events assuming the first null hypothesis is true. The area under the orange curve to the left of 0% gives a *p*‐value of 0.011 (Figure 4a). For this defined test, there is only a 1.1% chance that our observation of excessive earthquakes during the stressing periods can be explained by a model in which earthquakes are evenly distributed (temporally).

The random test predicts 0.0% ± 20.3% more earthquakes during the stressing period than the relaxing period (blue curve in Figure 4a) for the Ridgecrest area. Our comparison between the random probability function and the errorless observed number of earthquakes (gray dashed line in Figure 4a) gives a *p*‐value of 0.080. These two results of the two hypothesis tests are consistent with the alternative hypotheses that the declustered earthquakes are being influenced by the seasonally repeating nontectonic stress changes with >92% confidence.

However, the overlap of the two probability functions shows that the excessive earthquakes are not significantly different from random at the 92% confidence level. Although the excessive earthquakes during the stressing periods are different from random at more than the 61% confidence level, when uncertainties in the stress model are considered for both temporal randomness tests and for the excessive earthquake tests, randomness in time cannot be ruled out as a cause for the 25.3% ± 11.0% more earthquakes that occurred during the stressing period compared to the relaxing period. These tests are unfortunately associated with small numbers of events (*N* = 33).

In order to ensure that these statistical results obtained using the small sample number (Figure 4a) are not extremely sensitive to a small subset of the sampling, we ran the same bootstrapping exercise a hundred times (Figure S4). For each of the hundred bootstrapping runs, we randomly selected 30 earthquakes out of the total 33 events. This test reveals that we can hold the ∼2‐*σ* significance level for the first two hypothesis tests more than 50 times out of the total of one hundred test runs. For the worst result of these one hundred tests (15.0% ± 12.4% more earthquakes during the stressing period than relaxing period), we can still reject our first two null hypotheses with confidence levels of 89% and 76%, respectively.

Using the declustered strike‐slip earthquakes (*N* = 160) we perform the same three hypothesis statistical tests for the entire ECSZ‐WL transition zone. The first hypothesis test reveals that 6.4% ± 3.8% more earthquakes occurred during stressing periods, while the expected excessive number of earthquakes during these same stressing periods is 0%. The second random test predicts −0.0% ± 5.6% more events during stressing periods than those during the relaxing periods (Figure 4b), while the errorless observed number of excess earthquakes during stressing intervals (gray dashed line in Figure 4b) is 9.1%. These two tests yield *p*‐values of 0.045 and 0.052, respectively. Thus, we can reject the first two null hypotheses with ∼95% confidence level. The overlap of the probability functions, however, is ∼47%. Therefore, accounting for errors in the stress model for both the calculation of random occurrence in time and for the calculation of excessive earthquakes within the stressing intervals, we cannot rule out the possibility that a random process can explain the observations.

As mentioned earlier, we eliminate some of the declustered earthquakes that may have occurred on left‐lateral strike‐slip faults or those that may have been affected by poroelastic processes within the CGF region for the ECSZ‐WL transition zone. Including these eliminated earthquakes does not significantly change our statistical test results. If we use all the declustered strike‐slip‐faulting focal mechanisms (*N* = 227), then we obtain the excessive earthquakes of 6.3% ± 4.0% during the stressing periods. The random test predicts 0.0% ± 5.7% more events during the stressing periods compared to the relaxing periods. When we use less‐strictly‐eliminated declustered earthquakes (*N* = 184), the statistical test returns a similar result, which is 6.3% ± 3.8% more events during the stressing periods compared with relaxing periods (compare with Figure 4b). “Less‐strictly‐eliminated” means that, although CGF events are not included, we do include earthquakes that occurred near the intersections of the SNFZ and PVFZ (which both possesses a significant right‐lateral component) with the left‐lateral Garlock fault. Using these 184 earthquakes, our random test results in an expected −0.0% ± 5.6% more events during the stressing periods compared with relaxing periods.

For the ECSZ‐WL transition zone we performed the same statistical test including all of earthquakes from the declustered SCSN catalog (Zaliapin & Ben‐Zion, 2020) without using any selecting criteria, for reasons mentioned in Section 2.2. Using all the events within the transition zone for the last 13 years (*N* = 611), we found that the excessive earthquakes were 4.3% ± 2.4% during the stressing periods, while the random test predicts 0.0% ± 3.7% (Figure S5). These results are slightly less significant than those obtained using selected strike‐slip focal mechanisms only (Figure 4b), probably owing to the existence of normal and oblique earthquakes in the sample. Nonetheless, this new result still clearly reveals that more earthquakes have occurred during the stressing period than the relaxing period. Similar to the previous results, we can reject the first two null hypotheses with ∼95% confidence level, whereas the overlap is ∼47% (Figure S5).

**Figure 5:**
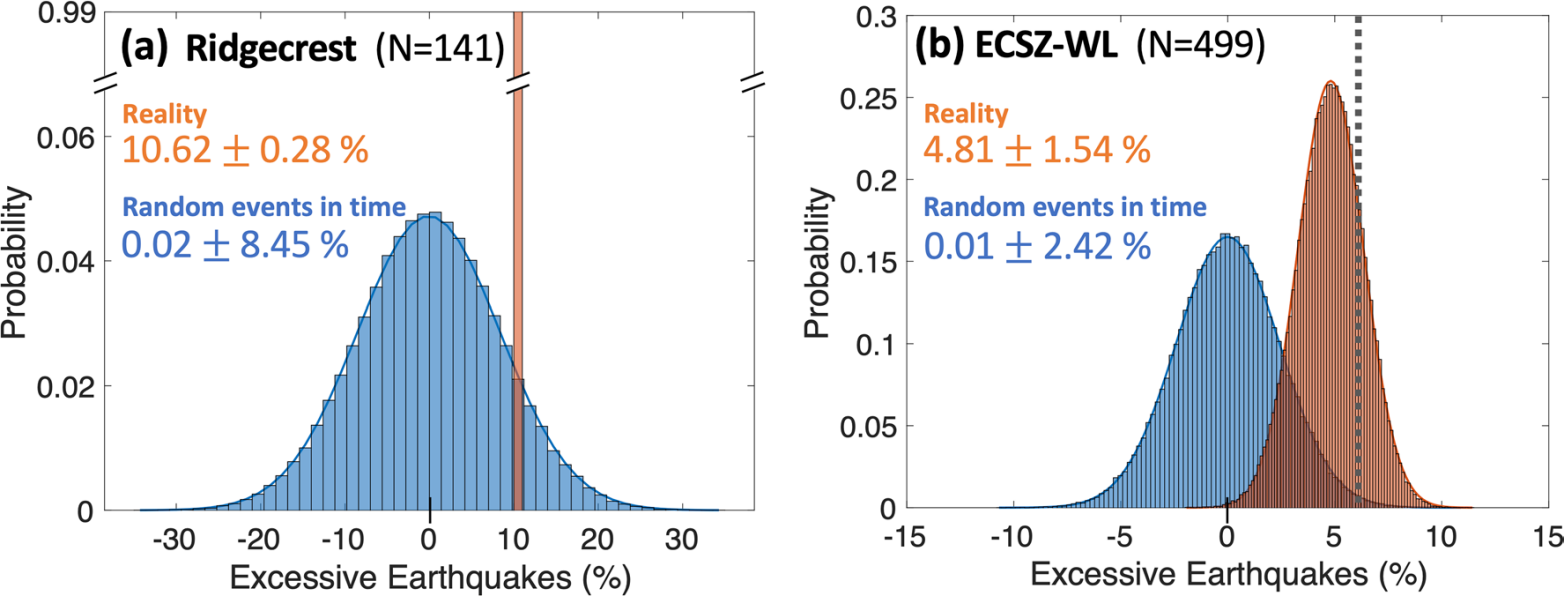
Statistical test results using the stacked total Coulomb stress change solutions (Movie S6) over the 13 years (except 2010). We assume that this stacked stress change 1‐year time series can represent all the years from 1981 to 2018 for this test. Probability Density Functions (PDFs) of declustered earthquake occurrences between January 1981 and December 2018 for the Ridgecrest area surrounding the epicenters of the 2019 Ridgecrest earthquake sequence (a) and for the entire ECSZ‐WL transition zone (b) shown in Figure 1. Each of these PDFs is plotted as a function of excessive number of earthquakes (%) during the stressing period. The blue curves are obtained by distributing declustered events in the original time of their occurrences. Mean values and standard deviations are presented for each PDF. The dotted gray vertical lines indicated the excessive number of earthquakes (%) obtained assuming our stress model is errorless (10.6% for [a] and 6.1% for [b]).

We next used the stacked monthly solution (Figure 2) to perform the same statistical tests, by assuming that this averaged annual Coulomb stress change pattern can represent the entire years between 1981 and 2018, which is regarded as a seismically active period for the ECSZ‐WL transition zone (Hauksson & Jones, 2020). Within the Ridgecrest area, this test (*N* = 141) reveals that 10.6% ± 0.3% more earthquakes occurred during the stressing periods compared to the relaxing periods (orange bar in Figure 5a), while the random test predicts 0.0% ± 8.4% more earthquakes during the stressing periods compared with the relaxing periods (blue curve in Figure 5a). These tests suggest that we can reject the first and second null hypotheses with confidence greater than the 99.9% and 90.0% intervals, respectively. For the third strict test, there is only 4.12% chance that the observations of excessive earthquakes during the stressing periods could be explained by a random process, after we take into account both the randomness of the timing of earthquake occurrences and the errors in our stress estimates.

For the ECSZ‐WL transition zone, based on the stacked monthly solution (Figure 2), we find 4.8% ± 1.5% more events during the stressing periods compared with the relaxing periods, while the random test only predicts 0.0% ± 2.4% excessive events during the stressing periods (Figure 5b; *N* = 499). We can reject the first and second hypotheses with confidence levels greater than 99% (*p*‐values of 0.001 and 0.003). However, we still have a large probability (∼21%) that our observations for the ECSZ‐WL transition zone resulted from a random process if we take into account both the randomness of the timing of earthquake occurrences and the errors in our stress estimates.

## 4 Discussion

### 4.1 Possible Dynamic Sources Responsible for the Seasonal Strain/Stress Anomaly Patterns

We show that horizontal nontectonic strain anomalies and the associated stress changes on right‐lateral strike‐slip faults repeat reliably in space and time across the ECSZ‐WL transition zone, including the Ridgecrest area. The exact dynamic source for the cGPS motions and therefore our kinematic model, however, remains enigmatic. We here briefly discuss possible sources that may contribute to the nontectonic seasonal strain/stress changes in the ESCZ‐WL transition zone.

Kim et al. (2021) showed that it is possible using cGPS data to extract short‐wavelength (tens of km), high‐amplitude horizontal strain anomaly patterns that are superimposed on long‐wavelength (hundreds of km) strain anomaly patterns in California and western Nevada. The long‐wavelength horizontal nontectonic strain solution can be explained as the Earth’s elastic response to the large‐scale hydrologic loading in the plate boundary zone (Kim et al., 2021). We also find that the short‐wavelength horizontal strain patterns (Figures S6a and S6b) across the ECSZ‐WL transition zone are superimposed on the long‐wavelength patterns (Figures S6c and S6d). For instance, the long‐wavelength nontectonic horizontal strain model for the Ridgecrest area predicts contractional dilatational strain during the early winter (December) and extensional dilatational strain during the early summer (June). The associated long‐wavelength stress changes also peak in May and June on the right‐lateral fault, although the magnitude is only up to 0.13 kPa. This long‐wavelength portion of the signal can only explain ∼8%–14% of the higher‐amplitude, shorter‐wavelength stress changes. The quantitative comparison between the long‐wavelength solution and its well‐determined dynamic source (hydrologic loading) suggests that a portion of seasonal strain anomalies inferred from cGPS data in the Ridgecrest area can be attributed to the large‐scale elastic response to surface loads. However, the short‐wavelength, high‐amplitude horizontal strain anomaly patterns cannot be explained by large‐scale hydrologic loading alone.

In addition to the large‐scale hydrologic loading, elastic and poroelastic responses to local hydrologic systems could explain some portion of the higher‐amplitude nontectonic signals inferred from horizontal cGPS data. Complicated mountain‐valley systems existing throughout the ECSZ‐WL transition zone (e.g., the Indian Wells Valley to the west of the 2019 Ridgecrest epicenters; the Searles Valley to the southeast of the epicenters; the Argus Range and the Panamint Valley‐Mountain to the northeast of the epicenters) may contain seasonal water distributions on and beneath the surface. These local hydrologic systems may significantly contribute to the seasonal strain anomaly patterns and the associated stress changes inferred from cGPS in the context of poroelasticity (e.g., Amos et al., 2014; Argus et al., 2005; Carlson, Shirzaei, Ojha, & Werth, 2020; Carlson, Shirzaei, Werth, et al., 2020; Chaussard et al., 2014; D’Agostino et al., 2018; González et al., 2012; Ji & Herring, 2012; N. E. King et al., 2007; Ojha et al., 2019; Silverii et al., 2019).

Another possible dynamic source responsible for the seasonal strain/stress variations is thermoelastic effects from the lateral gradients of temperature (e.g., Ben‐Zion & Allam, 2013; Prawirodirdjo et al., 2006). Horizontal temperature gradients across the High Sierra and nearby valleys are seasonally varying (Figure S7). A horizontal temperature gradient involving a 20°C temperature change over a wavelength of 120 km can produce horizontal surface strains of 2.5 × 10^−8^ (Ben‐Zion & Leary, 1986; Berger, 1975). The surface thermoelastic strains can be coupled into the underlying elastic crust and have a significant depth of influence related to the wavelength and amplitude of the temperature differences (see Section 4.2).

Lastly, seasonally varying barometric pressure and the associated surface load can also contribute to the seasonal stress changes. Gao et al. (2000) showed the ∼±1 kPa seasonal stress changes related to the seasonal variations in atmospheric pressure in the Long Valley Caldera near the ECSZ‐WL transition zone.

These suggest that thermoelastic strains, together with hydrologic loading and barometric loading, may explain a large portion of the observed seasonally repeating nontectonic anomaly patterns. For the complete explanation of the dynamics, it will be necessary to investigate each of the suggested possible sources and their relative amplitude and phases as Johnson et al. (2017b) did for northern California. The primary goal of this manuscript is to quantify the preseismic horizontal strain/stress anomaly patterns and investigate how, in combination with tectonic loading, they may influence seismicity rates in the Ridgecrest area and within the entire ECSZ‐WL transition zone. We leave rigorous investigations of the dynamics for future work.

### 4.2 The Accuracy of Our Assumptions in the Model

The no‐length‐change directions (strikes of vertical strike‐slip faults), upon which we calculate stress changes (King et al., 1994), are in accord with the orientations of strike‐slip focal mechanism solutions (Hauksson et al., 2012; Yang et al., 2012) and the surface trace of strike‐slip Quaternary faults (Jennings, 1994; Nicholson et al., 2017; Shaw et al., 2015) within the ECSZ‐WL transition zone (see Figure S17 from Kim et al. [2021]). A more complex geometry of the faults, however, will have an influence on the accuracy of our results. Our calculations assume that the strike‐slip faults in the ECSZ‐WL transition zone are vertical. This is probably a reasonable first order assumption for the horizontal Coulomb stress change calculations. By analyzing 618 declustered strike‐slip focal mechanism solutions (1981–2018; Hauksson et al., 2012; Yang et al., 2012; Zaliapin & Ben‐Zion 2020) we found that the minimum dip is 45.0°, and that only 6 of the 618 faults have dips between 45° and 50°, where a horizontal normal traction, originally assumed to be acting on a vertical fault, will be reduced by a factor of ∼30%. A total of 556 of the 618 focal mechanisms have dips larger than 65°, which can hold 90.6% of the estimated horizontal normal traction on a vertical fault (Figure S8). The horizontal shear traction, however, is not influenced by the variations in dip angles. In summary our assumption of vertical strike‐slip geometry for the Coulomb stress change calculations is reasonable because 90% of the events have dips that yield a 10% error or less in normal traction and only 1% of the events have dips that yield an error of 30% in normal traction.

In the ECSZ‐WL transition zone and the Ridgecrest area, many multistranded strike‐slip faults are conjugate (e.g., DuRoss et al., 2020). For the small events, it is challenging to distinguish whether they are occurring on right‐lateral or conjugate left‐lateral strike‐slip faults. Our assumption in the statistical tests is that all of the strike‐slip faulting focal mechanisms are on right‐lateral faults. Earthquakes that occurred on conjugate left‐lateral faults could perturb the significance of our statistical test results. We resolved Coulomb stress changes on left‐lateral strike‐slip fault orientations (another set of no‐length‐change directions that are generally perpendicular to corresponding right‐lateral strike‐slip faults). Within the Ridgecrest area, the Coulomb stress changes on left‐lateral faults (∼230°) also repeat reliably over years and they tend to be in phase with the right‐lateral Coulomb stress changes. In general, Coulomb stress on left‐lateral faults peaks in early summer (May and June) and it reaches a local minimum in December and January. Note that the styles of the stacked seasonal strain principal axes in the Ridgecrest area involve ∼E‐W extension during the summer and ∼E‐W contraction during the winter. The Coulomb stress changes on right‐lateral and left‐lateral faults are generally in phase because the seasonal ∼E‐W principal axes of extension and contraction produce nearly identical Coulomb stress changes for the respective strikes of 140° and 230° (Figure S9).

However, there are two exceptions in the seasonal strain patterns for the heavy precipitation years of 2017 and 2019. In these years the principal extensional axes for June rotate to an orientation of ∼230° (Figure S3). This orientation allows unclamping of right‐lateral faults during periods of positive extensional dilatation, but it has no impact on the Coulomb stress change for the left‐lateral faults. During the positive dilatation interval for May–June of 2017 and also for 2019, the other horizontal principal axis of strain involves a lesser amount of contraction, which acts perpendicular (clamps) to the left‐lateral fault plane (Figure S3). For 2017, the tectonic loading rate slightly cancels the negative seasonal rate on left‐lateral faults and the total stress change for May and June is slightly positive on these faults. For the heavy precipitation year of 2019, however, the total strain actually discourages slip on left‐lateral faults during May and June (Figure S9).

For the entire ECSZ‐WL transition zone that includes the Garlock fault (Figure 1), we performed statistical tests using the Coulomb stress change estimates on left‐lateral faults. These results reveal that there were 1.9% ± 3.3% more earthquakes during the stressing period on left‐lateral faults than the relaxing period, while the random test predicts 0.0% ± 4.7% more events during the stressing period (Figure S10). Within the 1‐sigma error level, our results do not support the main hypothesis that the rate of seismicity is correlated with the estimates of the total Coulomb stress changes resolved on left‐lateral faults. The overlap of the observed and randomly achieved probability functions is also larger than 75% (Figure S10). This result may suggest that the actual nontectonic stress loading in the ECSZ‐WL transition zone may not be optimally aligned (Hardebeck, 2020; Johnson et al., 2017b; King et al., 1994) to enhance seismicity on the left‐lateral strike‐slip faults, whereas these same non‐tectonic stress changes are more optimally oriented to influence seismicity on the right‐lateral strike‐slip faults.

Another source of uncertainty is the validity of our assumption that the horizontal stress/strain estimates at the seismogenic depths of 5–10 km are similar to the ones derived from surface observations. The depth of influence is dependent on both the source and wavelength of the nontectonic strain. The horizontal strain at seismogenic depths can be different from the strains inferred from the surface. Berger (1975) and Ben‐Zion and Allam (2013) show that the depth of influence within the elastic crust is of the order of the wavelength of the surface strains. In the case of the long‐wavelength hydrologic loading (100s of km), the strains at seismogenic depths of 5–10 km are indistinguishable from the surface strains associated with this loading (Kraner et al., 2018). Our nontectonic seasonal strain solution shows that the lateral wavelength is ∼50 km scale. For this wavelength, strains at the seismogenic depths of ∼10 km are expected to be ∼80% of the surface strain magnitude (Figure S11). We therefore conclude that the resolved seasonal nontectonic surface strains should have a substantial influence at seismogenic depth range of 5–10 km (Figures S8a and S11; Ross et al., 2019; Shelly, 2020).

### 4.3 Possible Triggering Mechanism of the 2019 Ridgecrest Earthquake Sequence

The 2019 Ridgecrest earthquake sequence consists of two large earthquakes: The M_w_ 6.4 event occurred first on the NE‐SW striking left‐lateral strike‐slip fault on July 4, 2019, followed by the M_w_ 7.1 event on a NW‐SE oriented right‐lateral strike‐slip fault on July 5, 2019 (e.g., Fielding et al., 2020; Xu et al., 2020). Although the majority of the seismic moment of the M_w_ 6.4 “foreshock” was released from left‐lateral ruptures, it has been reported that the initiating rupture of the M_w_ 6.4 foreshock may have been on a right‐lateral fault (e.g., Chen et al., 2020; Cortez et al., 2021; Huang et al., 2020; Lomax, 2020; Ross et al., 2019; Wang et al., 2020). Furthermore, studies have shown evidence that suggests the M_w_ 6.4 foreshock, at least, involved a complex rupture process on both NE‐SW and NW‐SE conjugate faults (e.g., Lee et al., 2020; Liu et al., 2020; Pollitz et al., 2020).

We do not consider dynamic or static stress changes during or following the M_w_ 6.4 and the M_w_ 7.1 events, although the magnitude of such stress changes must be orders of magnitude higher than the preseismic seasonal stress changes we present in this manuscript (e.g., Barnhart et al., 2019; Chen et al., 2020; Goldberg et al., 2020; Jin & Fialko, 2020; Lozos & Harris, 2020; Magen et al., 2020; Mancini et al., 2020; Qiu et al., 2020; Ramos et al., 2020; Toda & Stein, 2020; Wang et al., 2020). Our interest is rather to quantify and characterize preseismic nontectonic stress changes, which we argue in this manuscript may have provided preseismic stress increases on right‐lateral faults within the area of rupture.

Shown in Figure 3, we observe repeating seasonal nontectonic Coulomb stress changes of 0.623 ± 0.151 kPa within the Ridgecrest area (Figure 3). The seasonal stress changes on right‐lateral faults peak in every May and June between 2007 and 2019, including June of 2019, during the month prior to the 2019 Ridgecrest earthquakes. Our estimated tectonic stress loading rate in the Ridgecrest area is 1.4 kPa/yr. Using the preseismic stress change and the steady‐state loading rate (King et al., 1994), we find that the 2019 Ridgecrest earthquake sequence was advanced by 5.34 ± 1.29 months.

Similarly, Kraner et al. (2018) showed a possibility that the 2014 M_w_ 6.0 South Napa earthquake of August may have been triggered by nontectonic stress changes in South Napa region. They showed the average amplitudes of positive seasonal Coulomb stress changes peaked in every summer between 2007 and 2014 at 5.1 ± 1.6 kPa, including a month prior to the 2014 earthquake (within a 100 km^2^ area surrounding the epicenter of the 2014 earthquake). With their tectonic stress loading rate of 7.3 kPa/yr for the 100 km^2^ area (Kraner et al., 2018), we calculate that the M_w_ 6.0 South Napa earthquake was advanced by 8.38 ± 2.63 months.

If there were no seasonal stress changes, these 2014 and 2019 large earthquakes would have occurred whenever the accumulated tectonic stress reached the threshold failure level on the faults, regardless of seasons. However, the ratios of the nontectonic stress anomalies to the tectonic stress loading rates are large in both of the Ridgecrest and South Napa areas (∼0.5–0.7), suggesting that the nontectonic stress changes may have modulated the timing of the large events by a half a year. This suggests that, owing to these nontectonic stress changes, certain seasons may have an increased probability of rupture in large events (M_w_ ≥ 6) for regions where the ratios of the nontectonic stress anomalies to the tectonic stress loading rates are large.

The results of our statistical tests (with an implicit assumption of Coulomb failure criterion approach) also support this possible triggering mechanism for small earthquakes (M_w_ ≥ 2) for the Ridgecrest area and the entire ECSZ‐WL transition zone (Figures 4 and 5). On the other hand, for the same regions, we did not find any evidence supporting that seasonally modulated Coulomb stress rates are correlated with the rate of small earthquakes (M_w_ ≥ 2) within the 1‐sigma significance level (see Section 7 in Text S1; Figure S13). Instead, our findings suggest that earthquakes are more sensitive to absolute stress levels (stressing periods) than they are to the sign of the seasonally modulated stress rates.

We acknowledge that the real process of the seismic nucleation is more complicated than the simple Coulomb failure approach (e.g., Dieterich, 1994; Ellsworth & Bulut, 2018; Kato & Ben‐Zion, 2021; McLaskey & Lockner, 2014; Perry & Bendick, 2021; Yoon et al., 2019), and thus more rigorous investigations of the possible influences of seasonal stress rates on seismicity are still needed. Nonetheless, our results suggest that the seasonally repeating horizontal stress anomalies (stressing periods), within the respective formal uncertainties, are a feature of the horizontal field that should be taken into account when considering the linkage between stress changes and seismicity. There is a strong need to continue the monitoring using NOTA, together with other space geodetic constraints such as InSAR, to further reduce uncertainties in seasonal strain anomaly patterns and to more definitively define the impact on seismicity.

## 5 Conclusions

Using the position time series of cGPS data, we quantify horizontal nontectonic strain anomalies in California and find seasonally driven strain anomaly patterns in regions surrounding Ridgecrest, CA. The average principal axes of the nontectonic strain anomalies in May and June near the epicenters of the 2019 Ridgecrest earthquake sequence agree with the focal mechanisms of the 2019 events. The extensional strain principal axes usually align in the E‐W direction in May and June (Figure 2) and the magnitude of the extension is ∼2.5 × 10^−8^. The average principal axes of the strain anomalies in the December and January are opposite: contractional principal axes align in the E‐W direction with similar magnitudes to the May‐June extension. The associated seasonal Coulomb stress changes of ±0.623 kPa within the Ridgecrest area surrounding the 2019 Ridgecrest epicenters peak every May and June during the last 13 years (Figure 3), including June 2019, during the month prior to the 2019 Ridgecrest earthquake sequence. These seasonally repeating nontectonic stress changes may have advanced the timing of the 2019 events by 5.34 ± 1.29 months. This result suggests that the timing of the 2019 Ridgecrest earthquakes may have been modulated by nontectonic preseismic seasonal stress changes. After performing statistical tests, we reject our main null hypothesis that earthquakes occur regardless of seasonal stress changes within the ECSZ‐WL transition zone and the Ridgecrest area with confidence levels of 90%–95%. However, if we consider both the randomness of the timing of earthquake occurrences and the errors in our stress estimates at the same time, it is not possible to rule out that the observed excessive earthquakes that occurred during the stressing periods could be explained by earthquakes occurring randomly in time, regardless of season. Nevertheless, our statistical tests in general favor the hypothesis that small earthquakes from a declustered catalog (M_w_ ≥ 2; Zaliapin & Ben‐Zion, 2020) show modulation by the seasonal stress changes. The dynamics of the nontectonic seasonal strain/stress changes, however, remain enigmatic. We speculate that the driving force of the seasonal nontectonic signals may be a combination of the Earth’s crustal elastic (and/or poroelastic) responses to hydrologic sources, atmospheric loads, and thermoelastic responses to the lateral temperature gradients in the ECSZ‐WL transition zone. As such, further investigations of the dynamics are required. Furthermore, continued monitoring of the plate boundary zone using NOTA, along with other space geodetic methods, is vital for the continued advancement of our understanding of how seasonal stress changes influence seismicity.

## Supporting information

Supporting Information S1

Movie S1

Movie S2

Movie S3

Movie S4

Movie S5

Movie S6

Movie S7

## Data Availability

The cGPS position time series products, the declustered SCSN catalog and the focal mechanism solutions for this research are available in these in‐text data citation references: Blewitt et al. (2018), Zaliapin and Ben‐Zion (2020), Hauksson et al., (2012), and Yang et al. (2012).
