# Supplementary material for: Repeating Nontectonic Seasonal Stress Changes and a Possible Triggering Mechanism of the 2019 Ridgecrest Earthquake Sequence in California
Source: J Geophys Res Solid Earth. 2021 Sep 29;126(10):e2021JB022188. doi: 10.1029/2021JB022188 (PMC9285800; doi:10.1029/2021JB022188)
Supplement: Supplementary file 1 — Supporting Information S1 [file JGRB-126-0-s001.docx]

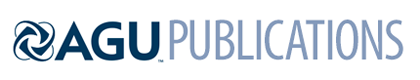


*Journal of Geophysical Research: Solid Earth*

Supporting Information for

**Repeating nontectonic seasonal stress changes and a possible triggering mechanism of the 2019 Ridgecrest earthquake sequence in California**

Jeonghyeop Kim^1^, William E. Holt^1^, Alireza Bahadori^1^, and Weisen Shen^1^

^1^ Department of Geosciences, Stony Brook University, Stony Brook, NY 11794

**Contents of this file**

Text S1

Figures S1 to S13

**Additional Supporting Information (Files uploaded separately)**

Movies S1 to S7

Text S1.

1. Treatment and inversion methods of cGPS data for continuous nontectonic horizontal strain anomalies

Here we provide a brief description of our methodology used for this manuscript. Readers are referred to Kim et al. (2021) and Kraner et al. (2018) for the full description in detail.

**1.1 The 4-month moving-time-window approach**

We analyze horizontal cGPS displacements over a 4-month time interval (Kraner et al., 2018). Kraner et al. found that the use of 6-month displacements did not guarantee to capture the peak-to-peak seasonal horizontal strain anomalies, owing to the fact that cGPS horizontal time series are often asymmetric rather than a perfect sinusoidal. The 4-month displacement is rather able to resolve the largest seasonal strain anomalies in South Napa regions (Kraner et al., 2018) as well as in regions much of southern California and western Nevada (Kim et al., 2021), and therefore the 4-month solution can be treated as the best solution that produces the peak-to-peak horizontal nontectonic strain. Kim et al. also quantified 1-month accumulated strain anomalies. Although this time interval is too short to capture full seasonal strain anomaly patterns, they showed that the integration of the 1-month solutions over 4 months generates identical spatiotemporal patterns to the 4-month accumulated strain solution (see Kim et al., 2021; Figure S18).

The 4-month time interval is treated as a moving-time-window, in which we quantify the evolution of the seasonal displacement and strain fields. For instance, the first 4-month time interval, where we obtain the first 4-month cGPS displacements, is between September 2006 and January 2007. We use the first 4-month displacements to quantify the associated 4-month strain field accumulated over the time interval. We then take a one-month time step to calculate the second 4-month strain field accumulated between October 2006 and February 2007. We repeat the same calculation taking one-month steps until we compute the last accumulated strain field between February 2019 and June 2019 (Kim et al., 2021). Note that here we analyze displacements and strains. Therefore, we label a strain solution accumulated over a 4-month time interval as a strain of the ending month.

**1.2 The inversion of cGPS displacements (4-month) for strain anomaly patterns**

Using the same inversion method described in Kraner et al. (2018) and Kim et al. (2021), we invert the horizontal 4-month displacements for each time step for a field of continuous horizontal displacements and strains on the surface of a sphere with formal posterior uncertainties (e.g., Beavan and Haines, 2001).

This inversion method involves a prior ‘steady-state tectonic’ strain rate model (see Kraner et al., 2018) derived from UCERF3 velocity consensus model (Field et al., 2014; Parsons et al., 2013). The UCERF3 velocity model was determined using multi-annual records of GPS data (>10 years) when there were no significant earthquakes (Field et al., 2014; Parsons et al., 2013). Therefore, we assume that the impact of tectonic transients on the UCERF3-driven ‘steady-state tectonic’ model is negligible (see the section 2.2.1 of Kraner et al., 2018). Multiplying a time-scale factor (4 months for this study) by the strain rate estimates, we determine the expected 4-month steady-state strain values within each of the 0.1º$\times$0.1º curvilinear grid cells across the ECSZ-WL transition zone (Figure 1). These prior steady-state strain values are jointly inverted with cGPS displacement observations, which are placed at the exact coordinates of each cGPS stations within the grid.

There is a trade-off in the inversion between fitting the prior steady-state strain estimates and the time-varying displacements of cGPS. To quantitatively show the goodness of fit to cGPS observations and the level of departure from the prior steady-state strain estimates in the joint inversion, we use a posteriori Standard Error of Unit Weight (SEUW), which is the square root of the reduced $\chi^{2}$ statistic divided by the number of degrees of freedom (Beavan and Haines, 2001; Kraner et al., 2018). For example, the small SEUW values correspond to a closer match to cGPS observations and strain fields that may involve large departures from the expected steady-state strain estimates (Kraner et al., 2018). The large SEUW values, on the other hand, represent large misfits to cGPS observations and smaller departure of the model strain fields from the expected steady-state strain estimates (Kraner et al., 2018).

An SEUW value of 1, in general, provides an ideal fit to GPS observations; however, an SEUW value of 1.5 is a realistic goal to achieve in the inversion due to uncertainties in GPS data (Beavan and Haines, 2001; Haines and Holt, 1993). To generate our time-dependent strain models presented here, we fit cGPS displacements with an average SEUW value (considering all time epochs) of ~1.5 (Kim et al., 2021) (Figure S6a, b). Kim et al. showed that it is possible to extract the large-scale, long-wavelength components of strain patterns whose time-dependent strain changes can be explained as the Earth’s elastic responses to the surface hydrologic loads (e.g., Argus et al., 2014) (Figure S6c, d). However, this later solution required a large SEUW value (6.37) implying that the long-wavelength strain solution filters out real shorter-wavelength, higher-amplitude components of strain signals captured by cGPS data (Kim et al., 2021) (Figure S6). For this study, we are interested in the high-amplitude, short-wavelength strain signatures in the Ridgecrest and ECSZ-WL transition zone regions, which represents an SEUW = ~1.5. Kraner et al. (2018) also settled for similar SEUW values of 1.6 – 1.8 for their cGPS-driven strain models in the regions near South Napa, California.

In order to decompose nontectonic strain anomalies from the inverted ‘total’ strain solutions, we subtract out the prior steady-state strain estimates. In other words, the departures of the inverted ‘total’ strain solutions from the prior steady-state strain are the nontectonic strain anomalies, which are captured by cGPS datasets (Kraner et al., 2018; Kim et al., 2021).

**2. Calculation of Coulomb stress changes associated with the transient strain anomalies**

We first convert our nontectonic horizontal strains into stresses, assuming that the crust is elastic and isotropic. We use the two Lamé Parameters: $\lambda$ of 0, which indicates incompressible elastic, and­­­ a shear modulus of 30 GPa (Kraner et al., 2018; Kim et al., 2021).

After this conversion, we quantify the associated Coulomb Stress changes resolved on fault geometries (King et al., 1994; Kraner et al., 2018). We compute the two possible no-length-change directions within the steady-state tectonic strain field to provide a continuous prediction of expected fault strikes (left-lateral and right-lateral) within each area (Holt and Haines, 1993; Holt et al., 2000). We use the prior steady-state strain estimates (Kraner et al., 2018; Kim et al., 2021) for this. In the ECSZ-WL transition zone, the two orthogonal no-length-change directions are consistent with the orientation of the major strike-slip faults (see Figure S17 of Kim et al., 2021). We resolve the nontectonic Coulomb stress changes as well as the steady-state tectonic stress loading rates (see the equations 5, 6, and 7 of Kraner et al., 2018) on the no-length-change directions, which agree with the strike of the right-lateral and left-lateral strike-slip faults in the ECSZ-WL transition zone including the Ridgecrest area. We assume the effective coefficient of friction as 0.4 throughout the region of analysis (e.g. Toda et al., 2005; Wang et al., 2020).

3. Random errors added to the time-dependent stress changes for the statistical tests

In order to consider the uncertainties in our stress estimates for the statistical tests (see the main text *Section 2.3.*) we calculate the excessive number of earthquakes 100,000 times for the 892 $\boldsymbol{k}\boldsymbol{m}^{\boldsymbol{2}}$ Ridgecrest area surrounding the 2019 Ridgecrest epicenters. For each of the calculations we add a set of random errors to the ‘errorless’ stress timeseries (over 150 months; Movie S1). Owing to the random errors that are added, the stress timeseries slightly varies within each bootstrap realization, and this leads to 100,000 different values for the excessive number of earthquakes.

To produce a time series that includes random errors, we generate 150 stress realizations (one for each month) that has added random errors that follow a normal distribution about the expected value with a 1-$\boldsymbol{\sigma= \pm}$ 0.15 kPa, obtained from the formal standard error of the stress estimates at each time within the 892 $\boldsymbol{k}\boldsymbol{m}^{\boldsymbol{2}}$ Ridgecrest area. This random error in time domain, $\boldsymbol{N(t)}$, is a white noise. Thus, we later apply a low-pass filter to account for more realistic temporal correlations. To do so, we first fit the total stress timeseries (Figure 3) with a sinusoidal function superimposed on a linear line in the least-squares sense. We assume the period of the sinusoidal function is a year. Second, we calculate the misfit between the model and the stress timeseries and then apply a Fast Fourier Transform (FFT) to the misfit. The misfit in the frequency domain, $\boldsymbol{M(\omega)}$, serves as a low-pass filter: we multiply $\boldsymbol{M(\omega)}$ by the white noise in the frequency domain $\boldsymbol{N(\omega)}$ and then take the inverse FFT to have $\boldsymbol{N}^{\boldsymbol{*}}\left( \boldsymbol{t} \right)$, which is the new random error time series. We finally adjust the amplitude of $\boldsymbol{N}^{\boldsymbol{*}}\left( \boldsymbol{t} \right)$ by multiplying by a value that is the 1-$\boldsymbol{\sigma}$ error in the stress time series (0.15 kPa) divided by the Root Mean Square between the $\boldsymbol{N(t)}$ and $\boldsymbol{N}^{\boldsymbol{*}}\left( \boldsymbol{t} \right)$ (0.5863 $\boldsymbol{\pm}$ 0.0643). We also perform the same procedure to add random errors to the time-dependent stress change estimates across the entire ECSZ-WL transition zone (Figure 1).

4. A vertical cross section of the elastic finite-element model

To quantitatively show the depth-dependent influence of the nontectonic surface strain field, we use the surface motions across the Ridgecrest strain anomaly (along the profile A-A' in Figure S6b) as boundary conditions in an elastic finite-element model (Figure S11). For this model we took the horizontal continuous displacement solution associated with the short-wavelength surface nontectonic strain solution of June 2019. We projected the horizontal surface displacements along the profile line A-A' onto the same profile line. This projected displacement field is the boundary condition on the top of the vertical cross section. We imposed rigid boundary conditions on the remaining three edges (right, left, and bottom). Based on these boundary conditions we computed the elastic finite-element model of strain (Figure S11). We also produced another vertical elastic half-space model using the same boundary condition on the top (the projected displacement field), but with freely deforming boundary conditions on the other edges. Both of the models confirm that the short-wavelength (~50 km) surface strain anomaly has a significant influence throughout the entire seismogenic thickness of up to 15 km for the Ridgecrest region (Figure S8a), including the estimated depths (~8-10 km) of the 2019 Ridgecrest earthquake sequence (*available at USGS website:* [*https://earthquake.usgs.gov/earthquakes/eventpage/ci38457511*](https://earthquake.usgs.gov/earthquakes/eventpage/ci38457511)*;* [*https://earthquake.usgs.gov/earthquakes/eventpage/ci38443183*](https://earthquake.usgs.gov/earthquakes/eventpage/ci38443183)*).*

5. The NLDAS-2 soil temperature estimates

The North American Land Data Assimilation System project phase 2 (NLDAS-2) (Xia et al., 2012) monthly VIC soil temperature model suggests that for a temperature anomaly of 120 km wavelength and amplitude of 20º C in June (Figure S7), the dilatational strains within the Ridgecrest area peak with values of 2.5×10^-8^ yr ^-1^ and have a depth of influence of ~50 % of surface strain magnitude at 15 km (Berger, 1975). This suggests that thermoelastic strains, together with hydrologic and atmospheric loading, may explain a large portion of the observed repeating seasonal anomaly pattern in the Ridgecrest region. Using NASA’s NLDAS-2 monthly soil temperature data and near-ground atmospheric temperature (surface to 2 m) as boundary conditions, one may model the time-dependent thermoelastic strain and stress field (Figure S7), which may lead to a possible link between the repeating, yet enigmatic, seasonal anomalies captured by cGPS and the surface temperature changes (thermoelastic loading). As mentioned in the main text, we leave rigorous investigations of the dynamics for future work.

**6. Sensitivity of our nontectonic strain estimates to a cGPS Station (TOWG)**

Our algorithm that identifies stations affected by poroelastic responses eliminates a cGPS station TOWG (Hudnut et al., 2015) in the vicinity (15.6 km northwest) of the epicenter of the 2019 M_w_ 7.1 Ridgecrest earthquake. This is due to the absence of its measurements during the severe drought period (2012-2015; see the main text section 2.1.). The measurements of the station are available for the period between August 2015 and May 2016 and between February 2017 and June 2019 for our preseismic horizontal strain analysis. We investigated the influence of this station on our nontectonic strain anomaly estimates, and thus, on the statistical test results. This investigation revealed that the influence is negligible. Incorporating the measurements of the station TOWG into other cGPS measurements does not change the overall features of the history of the nontectonic strain/stress patterns due to the short period of its availability. Even for the years between 2015 and 2019, we only found slight changes in our nontectonic strain estimates in detail. This subtle change leads to a slightly better statistical test results (Figure S12).

**7. A possible correlation between the rate of Coulomb stress changes and the number of declustered earthquakes**

In addition to use of the ‘stressing period’ for our statistical analyses, we investigated a possible correlation between the rate of Coulomb stress changes and the number of declustered earthquakes by using a ‘positive-stress-rate period’. We define the ‘positive-stress-rate period’ as time intervals in which a time step (for a month) has a value of Coulomb stress change greater than the immediately prior Coulomb stress estimate, but less than the next one.

We found that only ~33 % of the ‘positive stress-rate period’ overlaps with the ‘stressing period,’ for the Ridgecrest area. In other words, the overlap between the ‘positive stress-rate period’ and the ‘relaxing period’ is ~67 %. In the main text we showed more events appear to occur during the stressing periods than the relaxing periods (Figures 4 and 5; within 90-99 % confidence levels). Therefore, we should also expect fewer earthquakes during the positive stress-rate periods because ~67 % of these periods overlap with the relaxing periods, while only ~33 % of these periods overlap with the stressing periods.

As expected, for the Ridgecrest area using the 33 earthquakes, we obtained -7.9 $\pm$ 8.2 % of excessive earthquakes during the ‘positive stress-rate periods,’ while the random test gives 0.0 $\pm$ 16.6 % of excessive earthquakes during the same ‘positive stress-rate periods.’ We also performed the same statistical tests using the stacked solution with the 141 declustered earthquakes for the years between 1981 and 2018 for the Ridgecrest area. This test result reveals that ~0 % excessive earthquakes occurred during ‘the positive stress rate periods’ (Figure S13a). Furthermore, for the ECSZ-WL transition zone, we also found that fewer events occurred during the ‘positive-stress-rate periods’ than during the zero-stress-rate or negative-stress-rate periods (Figure S13b).

Statistically we cannot conclude seismicity rates are (negatively) correlated with the ‘positive stress-rate period’ because the statistical significance is less than the 1-$\sigma$ (Figure S13). However, this implies that we can accept the null hypothesis that earthquakes occur regardless of the seasonally modulated stress rates in the Ridgecrest area and the ECSZ-WL transition zone. On the other hand, we already showed a correlation between the number of earthquakes and the absolute stress levels (stressing periods) within 90-99 % significance levels. Therefore, we conclude that earthquakes appear to be more sensitive to absolute stress levels (stressing periods; Figure 4 and 5) than they are to the positive rate of the seasonally modulated stress changes (positive-stress-rate period; Figure S13) for the Ridgecrest area and the ECSZ-WL transition zone.

It is important to notice that the 4-month moving-time-window approach used in this manuscript generates centered moving averages of strain rates over each 4-month time window. That is, the actual monthly strain rates may be quite different from the smoothed strain rate estimates obtained using the 4-month velocity. Therefore, our statistical analyses between the ‘positive-stress-rate period’ and seismicity rates presented here in the supporting material may also be influenced by this smoothed solution, and further investigations are needed with finer strain rate solutions. For instance, Kim et al. (2021) pointed out that strain rate estimates based on 1-month time window may be more accurate than those based on a 4-month time window in representing monthly evolution of strain changes. We leave rigorous investigations of stress rates for future work. Nevertheless, we conclude that a comparison of these results with the much stronger link between excessive earthquakes and stressing periods suggests that earthquakes are more sensitive to absolute stress levels, or positive stressing intervals, in which such stress levels are situated within new highs.

**Figures S1 to S13**


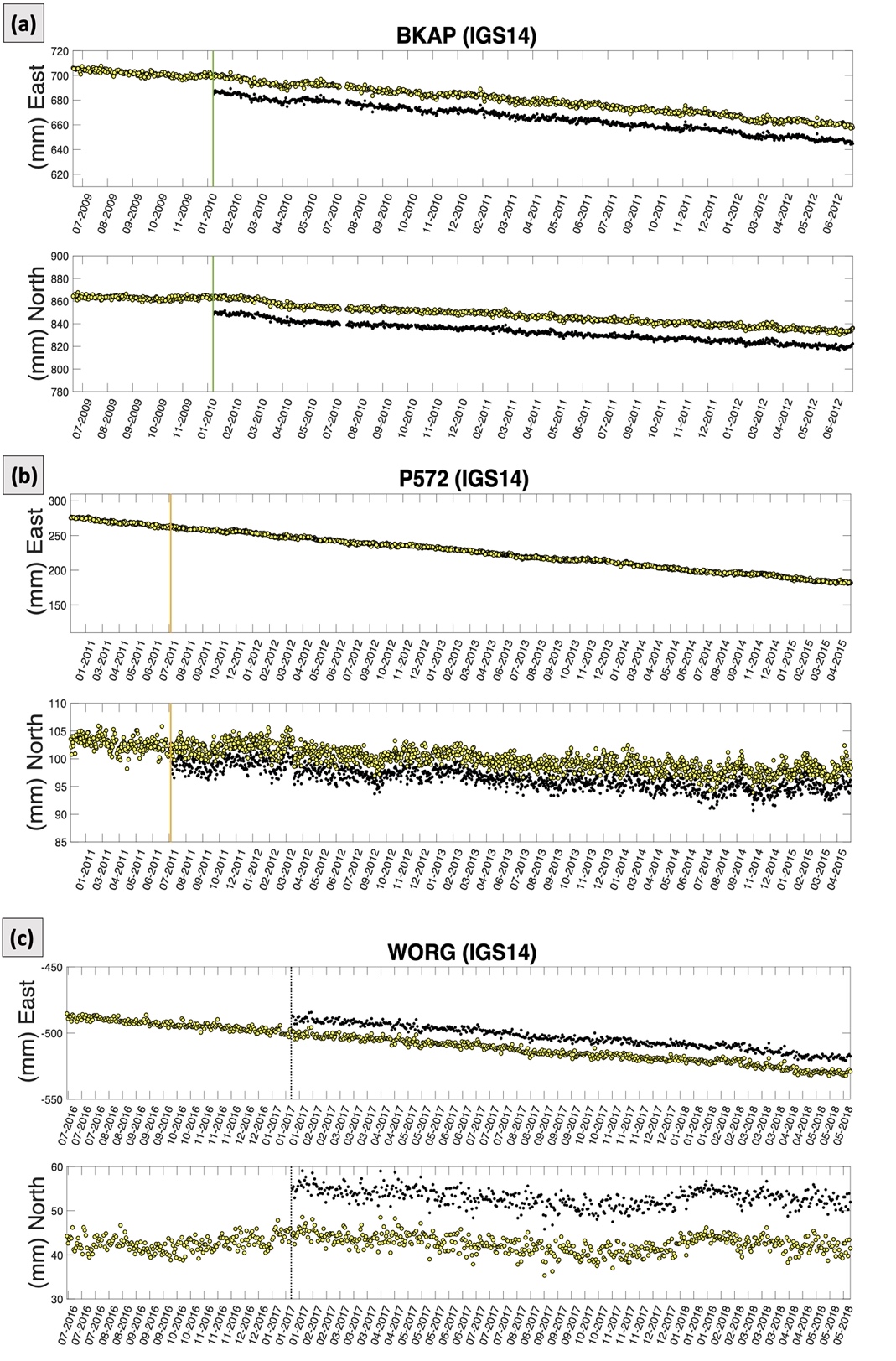


**Figure S1.** Three examples of offset-corrected position time series data from stations BKAP **(a)**, P572 **(b)**, and WORG **(c)**. The vertical solid lines in **(a)** and **(b)** indicate the timing of the equipment-related offsets from metadata provided by the Nevada Geodetic Laboratory. The dotted vertical lines in **(c)** indicate an unlisted offset that we corrected for the station WORG. Yellow solid circles are the corrected positions, and black circles are the original uncorrected positions after each of the discontinuous steps.


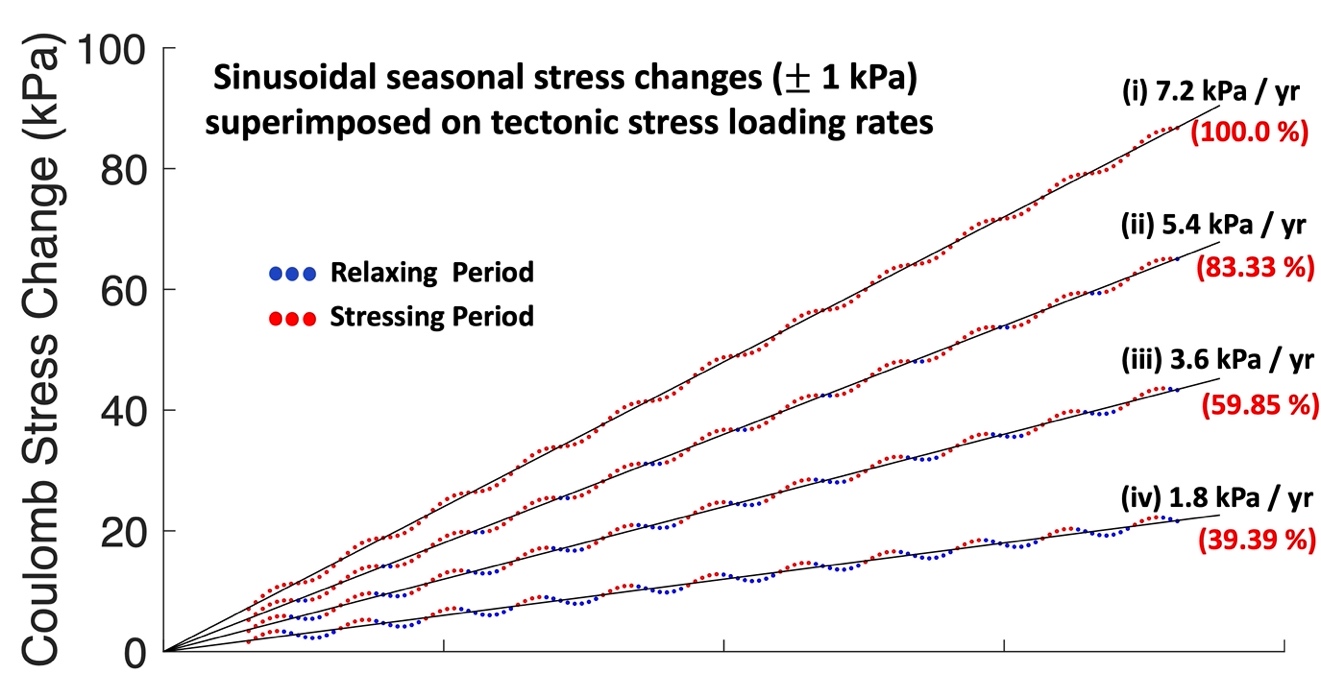


Figure S2. Model simulations of seasonal stress changes superimposed on different tectonic stress loading rates. A simple sinusoid is used to simulate a seasonal stress change of $\boldsymbol{\pm}$ 1 kPa that is superimposed on four different steady-state tectonic stress loading rates (black solid lines), labeled at the end of each time series (the x-axis is time, e.g., 150 months). Red dots indicate ‘stressing period’ months when each of the stress estimates is the maximum value compared to all the previous stress estimates; Blue dots show ‘relaxing period’ months when the stress estimates are not the maximum values compared to all the previous stress estimates. When the tectonic stress loading rates exceed 6 kPa/year, relaxing periods essentially disappear. This simulation demonstrates that the percentages of loading versus relaxing times of stress is dependent on the ratio of the magnitude of seasonal stress change to the magnitude of steady-state background tectonic loading. When seasonal stress changes are small in comparison to tectonic loading rates, then there may be very little influence of these seasonal stress changes on seismicity rates, owing to the lack of relaxing periods.

**Figure S3.** Principal axes of preseismic, nontectonic strain model for June inferred from cGPS data processed by Nevada Geodetic Laboratory. The red axes are extensional, and the gray axes are contractional. The background is dilatational strain (warm color is positive extensional dilatation). Selected 6 June solutions are presented for normal precipitation years of 2008 **(a)** and 2009 **(b)** (the 1^st^ column); drought years of 2013 **(c)** and 2014 **(d)** (the 2^nd^ column); and anomalously heavy precipitation years of 2017 **(e)** and 2019 **(f)** (the 3^rd^ column).


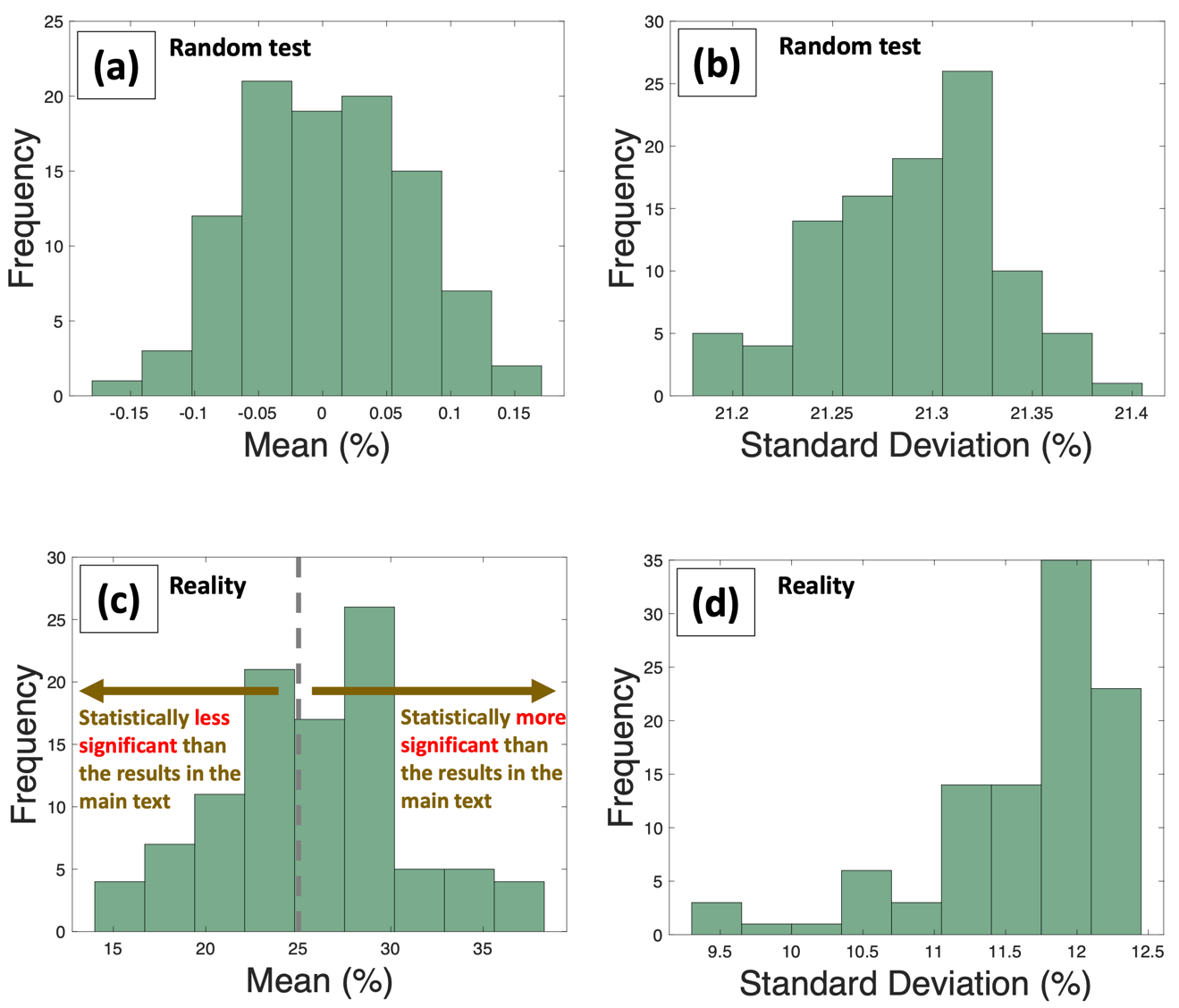


**Figure S4.** A sensitivity test of a bootstrapping exercise for the Ridgecrest area with one hundred randomly selected different subsets (N=30) of the 33 earthquakes. For each bootstrapping run, we obtain the two probability functions: one for the random test and another for reality (e.g., the blue curve and the orange curve in Figure 4a). We present a histogram of one hundred means of the random test **(a)** and a histogram of one hundred standard deviations of the random test **(b)**. We also present histograms of one hundred means of the reality test **(c)** and one hundred standard deviations of the reality test **(d)**. The random test is insensitive to a small subset of the sampling, while under sampling affects our reality test to some degree. The median value of the panel (c) is 26.9 % and the mean value is 26.1% with the standard deviation of 5.3 %. The minimum and maximum values are 15.0% and 37.8 %, respectively. The standard deviations of the reality test **(d)** is relatively insensitive to a small subset of the sampling compared to the means of the reality test **(c)**.


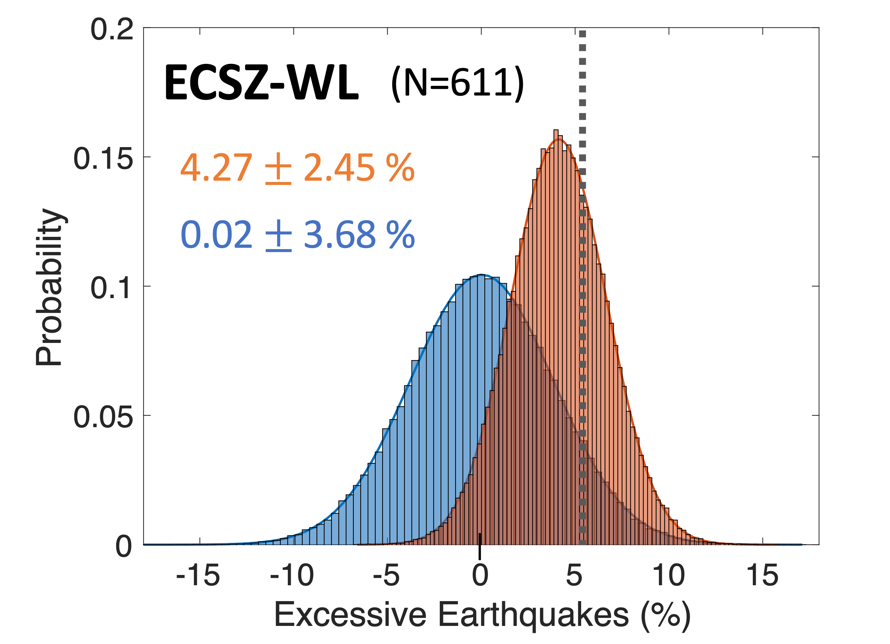


**Figure S5.** Statistical test results using the 13-year total Coulomb stress changes obtained using cGPS data. Probability Density Functions (PDF) of all of the 611 SCSN declustered earthquakes (Mw $\geq$ 2) between January 2008 and December 2018 within the ECSZ-WL transition zone shown in Figure 1. The PDF is plotted as a function of excessive number of earthquakes (%) during the stressing period. The blue curves are obtained by distributing all declustered events randomly in time, while the orange curves are obtained by distributing all declustered events in the original time of their occurrences. Mean values and standard deviations are presented for each test. The dotted gray vertical line indicates the excessive number of earthquakes (%) obtained assuming our strain/stress model is errorless (5.3 %).


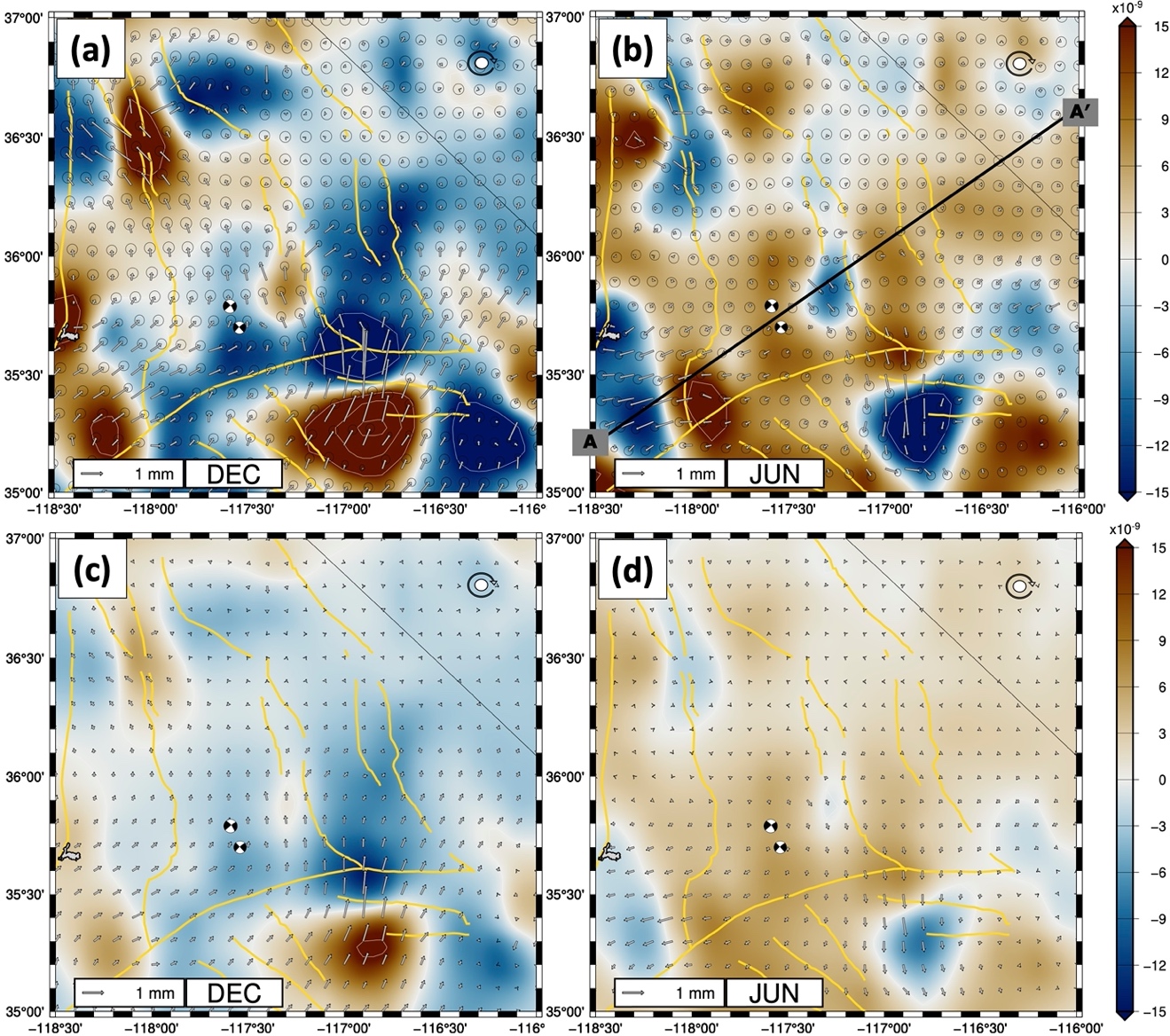


**Figure S6.** Averaged model displacements and dilatational strains over 12-13 years (2007-2019) for December **(a)**, **(c)** and June **(b)**, **(d)**. The displacement fields are relative to a local frame of reference indicated as a white circle surrounded by a circular arrow in Nevada. The background shows the dilatational strain fields background (red is extensional). We obtain these model fields from two different fits to seasonal components of cGPS data: an optimal fit to cGPS transients, with SEUW of 1.52, for **(a)** and **(b)** (short-wavelength, high-amplitude), and a smoothed fit to cGPS transient, with SEUW of 6.37, for **(c)** and **(d)** (longer-wavelength, lower-amplitude). Model error ellipses are 68 % confidence. We use cGPS data processed by Nevada Geodetic Laboratory (Blewitt et al., 2018). The A-A’ line indicates the profile for the vertical cross section of a homogeneous elastic half-space model presented in **Figure S11**.


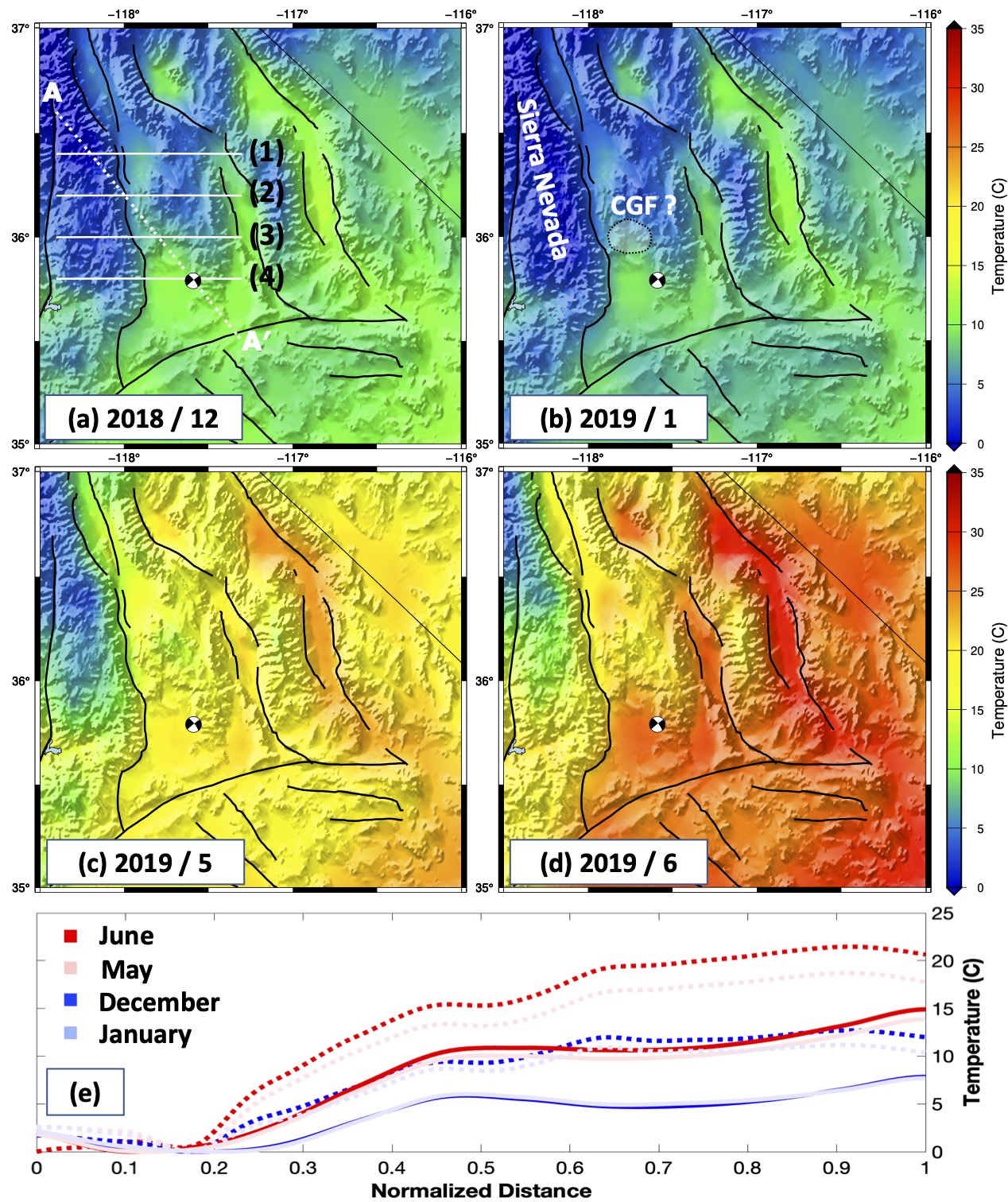


**Figure S7.** The NLDAS-2 monthly VIC soil temperature model at 0.125º intervals of latitude and longitude. VIC model uses three soil layers, whose thicknesses vary spatially. Noah-0.125º soil temperature model is also available for fixed thicknesses (0-2 meters). (both of VIC and Noah models are available at: <https://disc.gsfc.nasa.gov>; Mocko, 2014 and 2012). Soil temperature in Celsius degree for December 2018 **(a)**, January 2019 **(b)**, May 2019 **(c)**, and June 2019 **(d)**. Along the white dashed line A-A’ in **(a)** we obtain the horizontal temperature gradient profiles for the four months in **(e)** (colored dashed lines)**.** We also compute the average horizontal temperature gradients over the four white solid lines (1), (2), (3), and (4) in **(a)** for each of the four months and plot the profiles in **(e)** (colored solid lines)**.** These horizontal temperature gradient profiles **(e)** are normalized by subtracting their minimum temperature values to emphasize and only show the differences in the temperature gradient month by month. The shaded pattern is the SRTM15+ topography model (Tozer et al., 2019). The black solid lines indicate the major faults. The focal mechanism of the Mw 7.1 Ridgecrest earthquake is shown. CGF: Coso geothermal field.


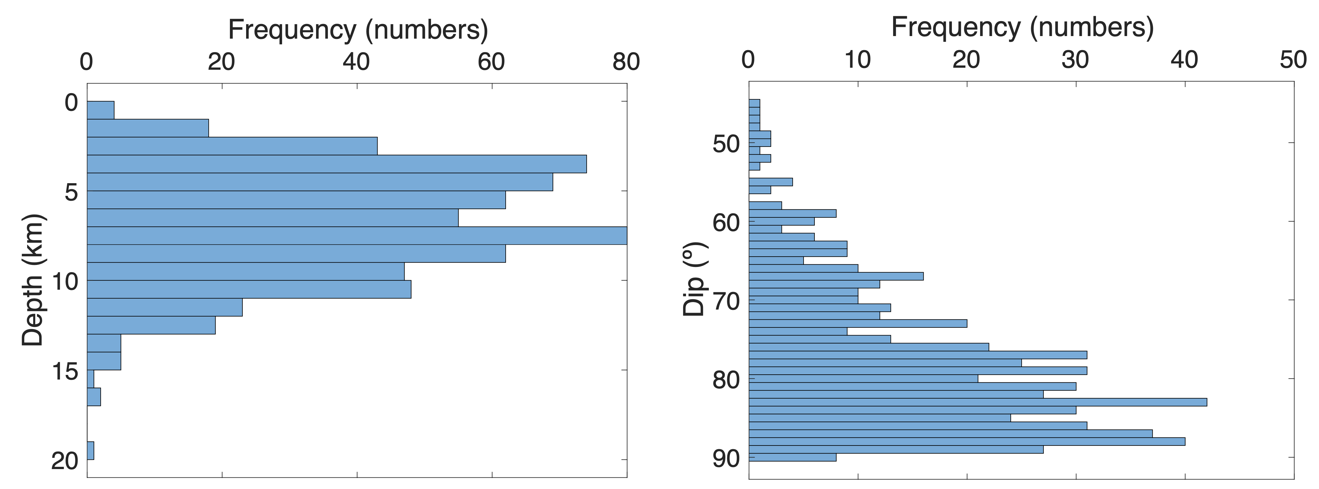


**Figure S8**. Histograms of the depths **(a)** and dips **(b)** of the declustered strike-slip earthquakes between January 1981 and December 2018 in the Ridgecrest region (Hauksson et al., 2012; Yang et al., 2012; Zaliapin and Ben-Zion, 2020). The mean depth is 6.77 km and the median is 6.69 km; the mean dip of the strike-slip faults is 77.89º and the median is 80.00º.


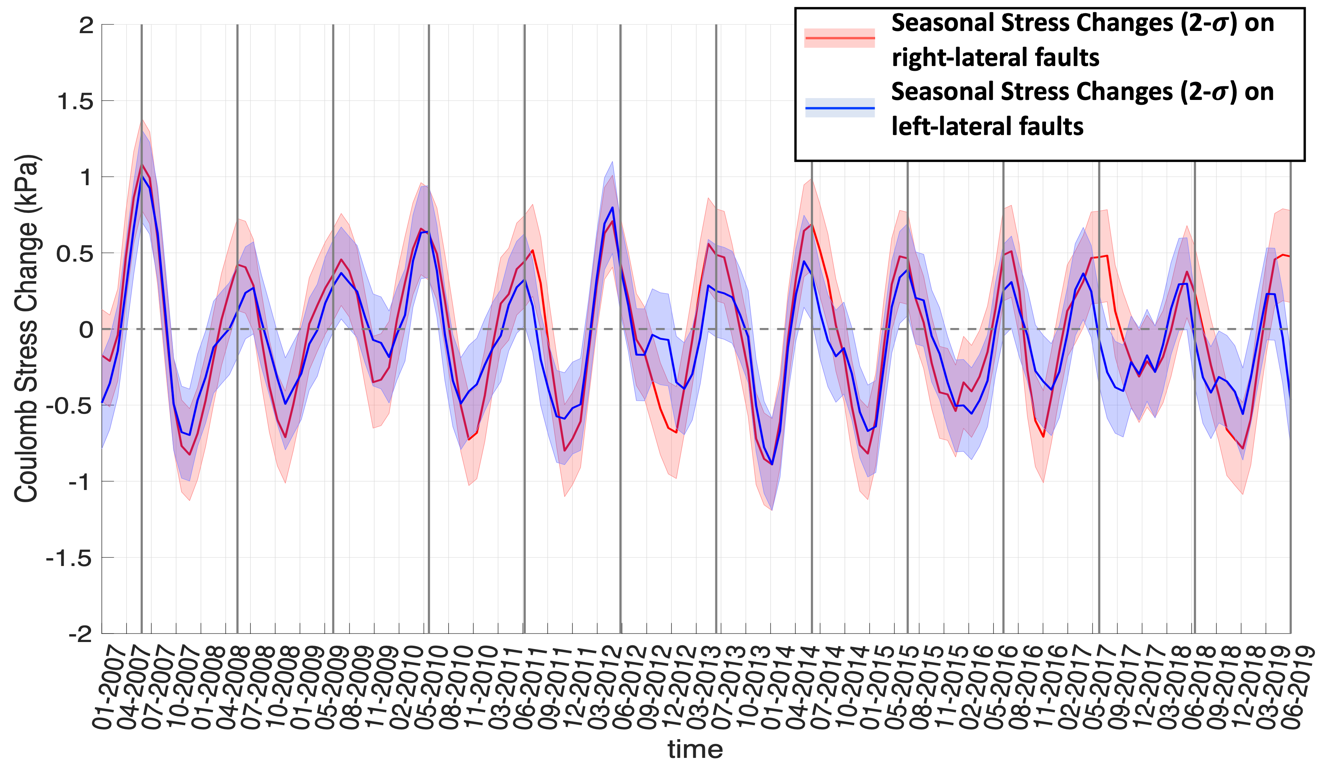


**Figure S9**. Two time series of nontectonic Coulomb stress changes resolved on right-lateral strike-slip fault (strike ~140º) (red) and left-lateral (strike ~230º) (blue) in the 892 $km^{2}$ Ridgecrest area surrounding the epicenters of the 2019 events. The shaded areas show 2 σ error level. Vertical gray lines indicate every June over the 13-year time period.


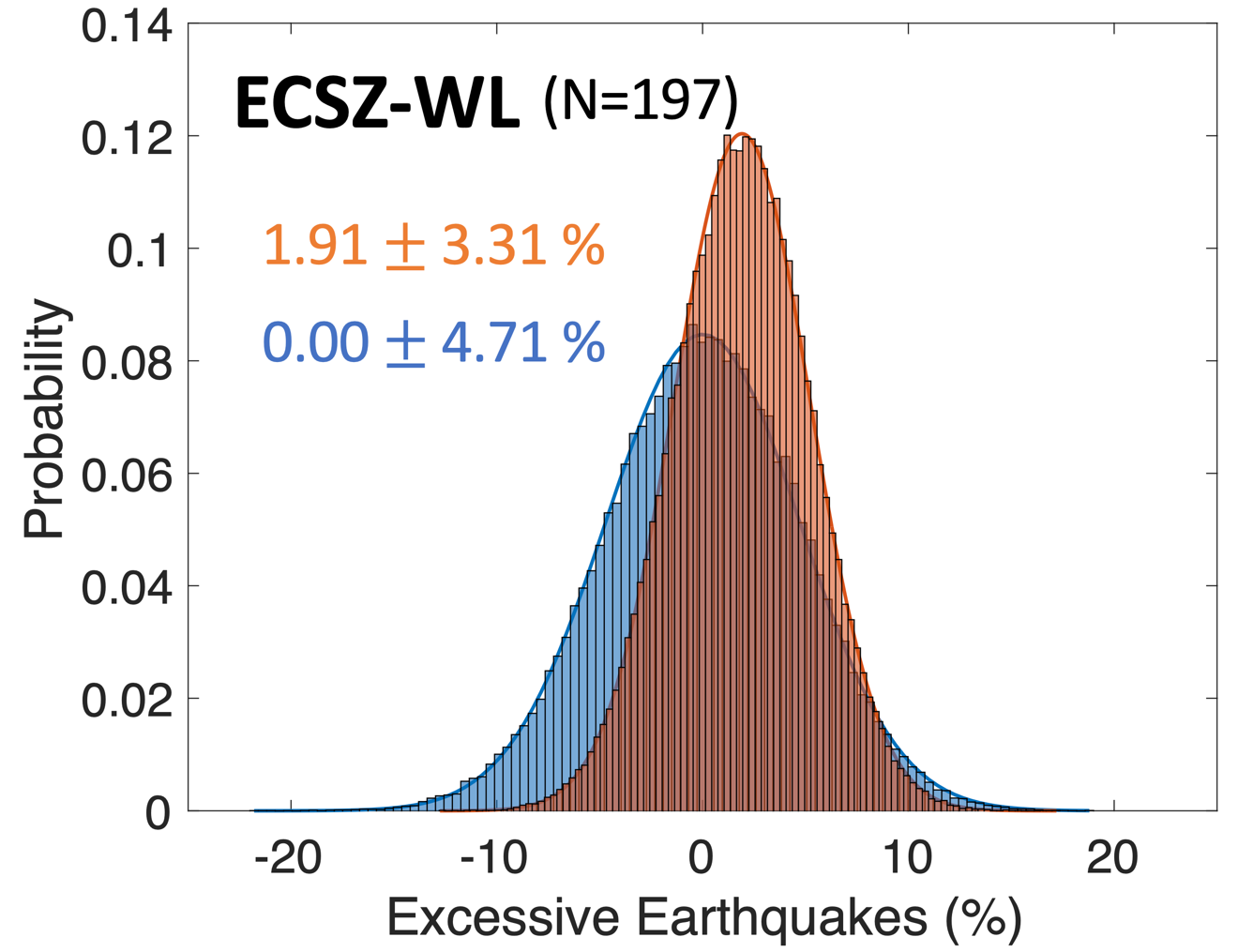


**Figure S10**. Statistical test results using the total stress changes resolved on left-lateral fault geometry for the Ridgecrest region shown in Figure 1. Two Probability Density are plotted as functions of excessive number of earthquakes (%) during the stressing period. The blue curves are obtained by distributing earthquakes randomly in time, while the orange curves are obtained by distributing the events in the time of their occurrences. Mean values with standard deviations are presented for each of the tests. The number of declustered strike-slip earthquakes is 197. We exclude 30 events that occurred near the CGF area (227-30=197).


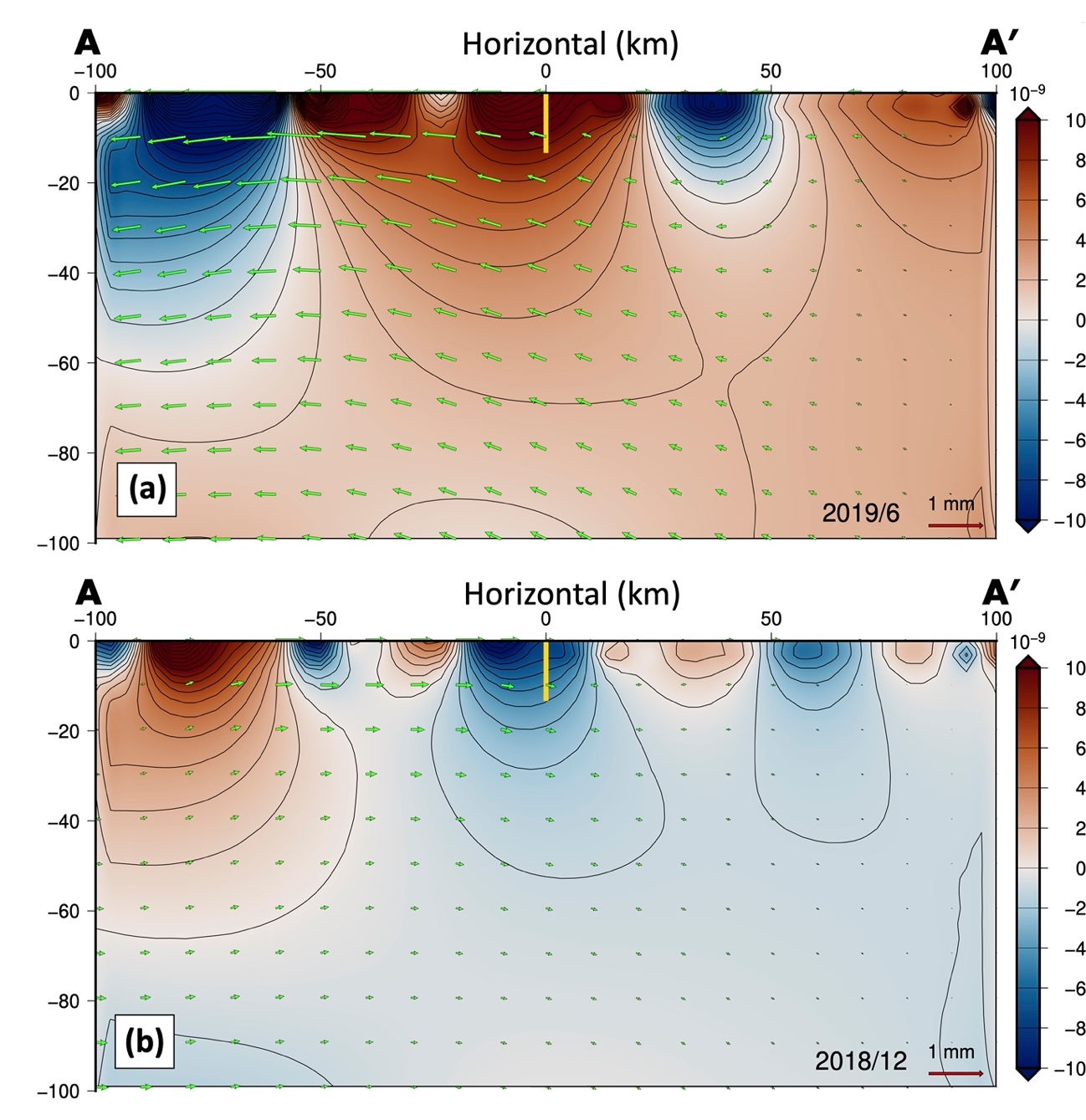


**Figure S11**. Vertical cross sections of a homogeneous elastic half-space model for June 2019 **(a)** and December 2018 **(b)**. We use surface displacements along the profile A-A’ presented in Figure S6b as surface boundary conditions to solve the elastic model. We take the surface horizontal displacements in Figure S6b projected into the profile A-A’ and reframe them with respect to the point A’. The reason for this reframing is because we set the vertical boundary (right) as a rigid boundary. The background is model dilatational strain (red is extensional) obtained from the elastic model. The goal of this simple model is to illustrate the depth of influence of the strain anomalies observed on the surface in the elastic lithosphere. Results show significant depth of influence at seismogenic thicknesses (5-10 km) in the Ridgecrest area. The Ridgecrest fault is plotted as a yellow line (with the length of 15 km), assuming the fault is vertical.


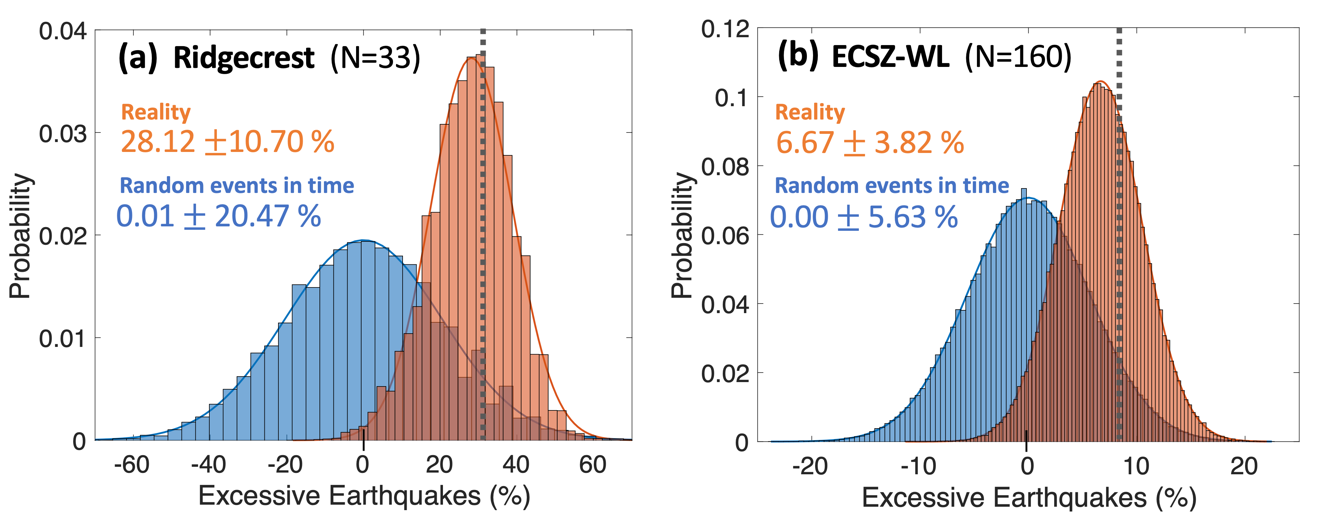


**Figure S12.** Statistical test results using the 13-year total Coulomb stress changes obtained using cGPS data (including additional measurements of station TOWG)**.** Probability Density Functions (PDF) of declustered earthquake (Mw $\geq$ 2) occurrences between January 2008 and December 2018 for regions surrounding the epicenters of the 2019 Ridgecrest earthquake sequence (the 892 $km^{2}$ area) **(a)** and for the ECSZ-WL transition zone shown in Figure 1 **(b)**. Each of these PDF is plotted as a function of excessive number of earthquakes (%) during the stressing period. The blue curves are obtained by distributing all declustered events randomly in time, while the orange curves are obtained by distributing all declustered events in the original time of their occurrences. Mean values and standard deviations are presented for each test. The dotted gray vertical lines indicate the excessive number of earthquakes (%) obtained assuming our strain/stress model is errorless (31.7 % and 8.3 %).


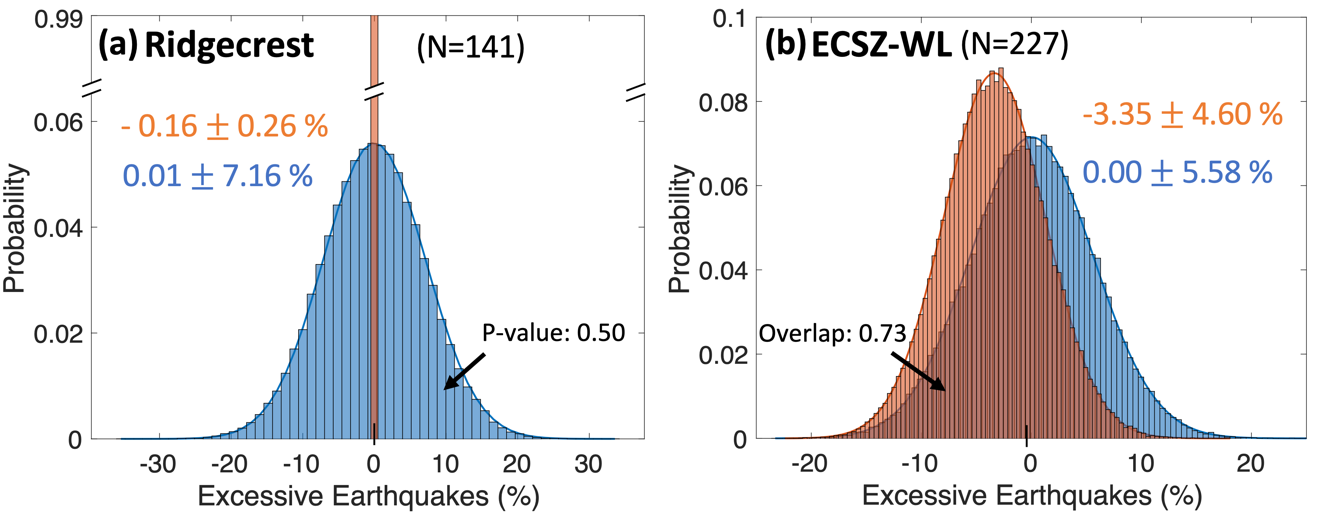


**Figure S13.** Statistical test results with the ‘positive-stress-rate period’ (see section 7 in this supporting text). Probability Density Functions (PDF) of declustered earthquake (Mw $\geq$ 2) for the 892 $km^{2}$ Ridgecrest area **(a)** and for the ECSZ-WL transition zone **(b)**. Each of these PDF is plotted as a function of excessive number of earthquakes (%) during the positive-stress-rate period. The blue curves are obtained by distributing all declustered events randomly in time, while the orange curves are obtained by distributing all declustered events in the original time of their occurrences. Mean values and standard deviations are presented for each test.
